# Gene body methylation buffers noise in gene expression in plants

**DOI:** 10.1093/nar/gkag127

**Published:** 2026-02-17

**Authors:** Jakub Zastąpiło, Robyn A Emmerson, Liudmila A Mikheeva, Marco Catoni, Ulrike Bechtold, Nicolae Radu Zabet

**Affiliations:** School of Life Sciences, University of Essex, Colchester CO4 3SQ, United Kingdom; School of Biosciences, University of Birmingham, Birmingham B15 2TT, United Kingdom; Department of Medical and Molecular Genetics, School of Basic and Medical Biosciences, King’s College London, London SE1 9RT, United Kingdom; School of Biosciences, University of Birmingham, Birmingham B15 2TT, United Kingdom; School of Life Sciences, University of Essex, Colchester CO4 3SQ, United Kingdom; Department of Biosciences, Durham University, Durham DH1 3LE, United Kingdom; School of Life Sciences, University of Essex, Colchester CO4 3SQ, United Kingdom; Blizard Institute, Barts and The London School of Medicine and Dentistry, Queen Mary University of London, London E1 2AT, United Kingdom; Centre for Epigenetics, Queen Mary University of London, London E1 2AT, United Kingdom

## Abstract

Non-genetic variability in gene expression is an inevitable consequence of the stochastic nature of processes driving transcription and translation. While previous studies demonstrated that gene expression noise is negatively correlated with gene body methylation, the function of this correlation remains poorly understood in multicellular systems. Here, we provide a first functional link between gene body methylation and transcription noise in plants. We investigated a mutant with partial loss of CG methylation (*met1-1*) and 10 epigenetic recombinant inbred lines (epiRILs) generated by a cross between Col-0 and *met1-3* plants, and observed an increase in gene expression noise, but this was not the case in *met1-3* with complete loss of CG methylation. Loss of CG methylation in *met1-3* could be compensated by a low but significant gain of non-CG methylation that buffers the noise in gene expression. Overall, our results show that gene body methylation has a functional role in reducing variability in transcription in a large subset of housekeeping genes, which require precise expression patterns to meet metabolic requirements. Genes lacking this noise-buffering effect are mainly enriched in stress response, where variability in gene expression can be seen as highly beneficial.

## Graphical Abstract

**Graphical Abstract:**
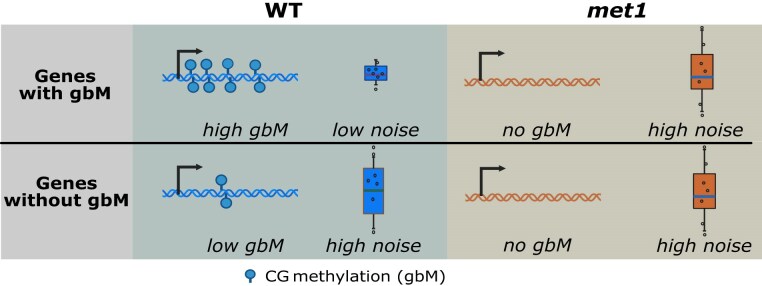

## Introduction

Genetically identical organisms grown under indistinguishable conditions may display different levels of gene expression. This difference or noise in gene expression is ubiquitous across biological systems and may lead to functional alterations between individuals [1]. Previous work has shown that transcriptional noise (variability) between genetically identical individuals can arise from the stochastic nature of the molecular processes influencing transcription and translation [2]. In animals, gene expression variability is thought to play a role in immune responses [3] and, in yeast, it is linked to improved survival under stress [4]. In plants, gene expression variability is present in the form of seed germination times, where it is a part of a bet-hedging strategy of germination [5, 6]. More generally, gene expression noise is relatively widespread in plants, and it has been proposed that it could drive developmental patterns and may be important in stress responses [2, 7, 8]). Recent advances in real-time monitoring of transcription initiation have shown that the extensive contribution of intrinsic noise at the cell level determines tissue-level responses especially under environmental stress conditions such as heat and phosphate starvation [9, 10]. Our understanding of how noise affects inter-plant variability and the mechanisms controlling this noise is limited. A previous study suggested that highly variable genes are smaller, are potentially targeted by a higher number of transcription factors (TFs), and have a more compacted chromatin environment [2].

Thus, a major source of variation is likely to be linked to epigenetic changes which are also responsible for different types of cellular memory, including stress memory [11]. Epigenetic changes lead to altered chromatin structure which has been linked to gene expression noise in isogenic cell populations [12]. For example, in Arabidopsis, overexpression of CHR23, a chromatin-remodelling ATPase, increased gene expression variability in a distinct subset of genes linked to environmental stress, resulting in growth variability [13]. Similar effects have been identified in yeast cells with altered histone deacetylase (HDAC) activity [14].

In *Arabidopsis thaliana*, DNA methylation is a heritable epigenetic mark that affects gene regulation [15]. Cytosine methylation can be subdivided based on the sequence context into mCG (CG), mCHH (CHH), and mCGH (CGH). Gene body methylation (gbM) consists of an enrichment of CG methylation within the transcribed regions of a gene and a depletion at the transcriptional start and termination sites together with an overall depletion of CHG and CHH (where H stands for A, C, or T) within the gene [16]. Methylation in CG, CHG, and CHH contexts has different maintenance pathways, but these are not completely independent and there is a link between maintenance of CG and non-CG methylation. In particular, CG methylation at gene bodies triggers accumulation of CHG methylation [17], but IBM1 (INCREASE IN BONSAI METHYLATION 1) demethylase removes this [18]. Methylation in the CHH and CHG context is linked to the transcriptional silencing of transposable elements (TEs) through the RNA-directed DNA methylation (RdDM) pathway (reviewed by Matzke *et al.* [19] and Fultz *et al.* [20]), and CHH and CHG methylation in genes and promoter regions is also linked with gene silencing [21].

In plants, there are four types of genes with respect to DNA methylation: (i) genes with gbM (CG methylation present and non-CG methylation absent); (ii) genes with TE-like methylation (non-CG methylation present and/or CG methylation); (iii) genes with promoter methylation (CG and/or non-CG methylation in the gene promoter); and (iv) unmethylated genes (lacking both CG and non-CG methylation). While the TE-like methylation has been linked with silencing of gene expression [19, 20], it is still not clear why some genes have gbM while others do not. Previous studies have shown that the presence of gbM corresponds to higher gene expression compared with its absence [22]. Furthermore, it has been speculated that gbM is associated with reduced transcriptional noise or erroneous transcription [21, 23, 24], and a recent analysis of single-cell RNAseq data of the root quiescent centre cell suggested that gbM is involved in decreasing erroneous transcription by reducing intron retention and lowering transcription noise compared with unmethylated genes [25].

Gene expression variability can be measured under constant [26] or changing conditions [27]. In this study, we investigated the tissue-level gene expression variability in *A. thaliana*, focusing both on intra-plant variability under changing conditions (considering different time points) and on inter-plant variability under constant conditions (different biological replicates). First, we identified genes with high expression variability across different time scales and biological replicates and found a correlation between gene expression variability at the tissue level and DNA methylation. Most importantly, we then investigated gene expression variability under constant conditions (across biological replicates) in several DNA methylation-deficient mutants and epigenetic recombinant inbred lines (epiRILs), generated by a cross between the wild-type Columbia-0 (Col-0) and *met1-3* plants, and found that loss of DNA methylation in gene bodies (and in some cases in promoters) leads to an increase in gene expression variability.

## Materials and methods

### Datasets

First, we used two publicly available sets of microarray mock expression data: (i) mock drought series [28] (GSE65046) with four biological replicates at each time point and measurements performed every day for 14 days (resulting in 56 samples; discovery dataset) and (ii) mock data from a high light series [29] (GSE78251) with four biological replicates at each time point and measurements performed every 30 min for 6 h (52 samples, validation dataset). In both series, tissue from *A. thaliana* leaf 7 of a separate plant for each biological replicate was harvested and sequenced using CATMA microarrays [30]. The plants in both series were 5-week-old plants grown in the same controlled-environment room under 8 h/16 h light/dark conditions 21°C, 65% relaive humidity at 150 μmol m^−2^ s^−1^ at the start of the measurement series.

The differences between both datasets were the sampling times. For the discovery dataset, one sample per day was harvested in the middle of the light period over a duration of 14 days, while for the validation dataset, the sample was harvested every 30 min for 6 h across the 8 h light period, starting 2 h into the light period.

The two datasets used CATMA microarray, and a consensus probe set was constructed, featuring only probes present in both [31] datasets (Supplementary Fig. S1). The combined datasets were normalized together, using the cyclic LOESS normalization method [31]. It is worth noting that one limitation of these microarray data is that they are limited to the investigation of splicing alterations [32].

For DNA methylation, we used publicly available bisulfite-converted sequencing data for 3-week-old leaves harvested from the wild-type Col-0 ecotype [33, 34]. In addition, we used bisulfite-converted sequencing data for 2-week-old leaves harvested from wild-type Col-0 Arabidopsis, *met1-1*, and *met1-3* from Catoni *et al.* [35]. The whole-genome bisulfite sequencing (WGBS data) was processed following the protocol outlined by Catoni *et al.* [36].

### Plant growth and library preparation

Seeds of epiRILs [37] and mutant alleles *met1-1* [38] and *met1-3* [39] and their corresponding Col-0 plants were stratified at 4°C for 4 days and plants were grown in half-strength Murashige and Skoog (1⁄2 MS) 1.5% agar vertical plates. Plants were grown under long-day conditions (21°C, 16 h light, 8 h dark). About 15–20 seedlings were flash frozen in liquid nitrogen (150 mg per sample). Total RNA was isolated from seedlings using the RNeasy Plant Mini Kit (Qiagen), following the manufacturer’s instructions. The RNA quality and integrity were assessed on the Agilent 2200 Tape Station. Libraries for RNA expression analysis were prepared in triplicate from 1 µg of high-integrity total RNA (RIN > 8) using the TrueSeq Stranded mRNA Sample Prep Kit (Illumina, San Diego, CA, USA) following the manufacturer’s instructions. Library quality and fragment sizes were checked with a TapeStation 2200 (Agilent Technologies, Santa Clara, CA, USA) instrument. Bisulfite-converted DNA libraries of epiRILs, together with wild-type Col-0 control for genomic sequencing were performed starting from 0.5–1 μg of genomic DNA using the NEBNext DNA Sample PrepReagentSet1 (New England Biolabs), following the Illumina Genomic Sample Prep Guide (Illumina), as previously described [40]. The samples have been sequenced with Illumina NextSeq 500 instruments, generating paired-end reads. All datasets generated or publicly available that were used in this study are listed in Table 1.

**Table 1.** Datasets

Type	Method	Condition	Accession	Source
DNA methylation	WGBS	epiRILs	GEO/GSE255302	Generated in this study
Gene expression	RNA-seq	epiRILs	GEO/GSE255301	Generated in this study
Gene expression	RNA-seq	Col-0	ENA/E-MTAB-16182	Generated in this study
Gene expression	RNA-seq	*met1-1*	ENA/E-MTAB-16182	Generated in this study
Gene expression	RNA-seq	*met1-3*	ENA/E-MTAB-16182	Generated in this study
Gene expression	Microarray	Discovery—mock drought	GEO/GSE65046	[41]
Gene expression	Microarray	Validation—mock high light	GEO/GSE78251	[42]
DNA methylation	WGBS	Col-0	GEO/GSE38286	[43]
DNA methylation	WGBS	Col-0	GEO/GSE89592	[40]
DNA methylation	WGBS	*met1-1*	GEO/GSE89592	[40]
DNA methylation	WGBS	*met1-3*	GEO/GSE89592	[40]

### Genomic data analysis

Sequenced reads were processed with Trimmomatic (version 0.38) [44], with the parameter ILLUMINACLIPTruSeq3-PE.fa:2:30:10:2:True, LEADING:3 TRAILING:3 SLIDINGWINDOW:4:15 MINLEN:36, to remove adapters and discard low-quality reads. The parameter HEADCROP:10 was used on WGBS data to remove guanine-biased calls at the 3′ region of paired reads. The cleaned reads were mapped to the Arabidopsis reference genome (TAIR10 version) using HISAT2 (version 2.2.1) [45] (default parameters) for RNA-seq and Bismark (version 0.24.02) [46] for WGBS datasets, with the parameters -N 1 -L 20 -p 4 -X 1000 -score_min L,0,-0.6.

For RNAseq, samtools (version 1.9) was used to generate bam files, and mapped reads were subsequently counted using htseq-count (version 2.0.2) [47], with the following parameters: –order = pos –mode = union –nonunique = none –type = gene –idattr = gene_id. Then, the raw count per gene was used to determine differentially expressed genes between tested condition using DESeq2 (version 1.40.2) [48] with default parameters and design formula of ∼ Condition. Briefly, size factors were estimated using the median-of-ratios method, dispersion was estimated using the parametric fit-type, and the significance of coefficients in a negative binomial general linear model (GLM) was tested for. Gene-wise *P*-values were adjusted using the Benjamini–Hochberg procedure, and genes with false discovery rate (FDR)-adjusted *P*-values <0.05 and log2 fold change (FC) expression of < −1 or >1 were treated as differentially expressed. To ensure that library size does not affect the estimation of the coefficient of vatiation (CV) for the RNA-seq in Col-0, *met1-1* and *met1-3*, we down-sampled the libraries to 9 million reads (which was the smallest library).

For WGBS, mapped reads have been de-duplicated with deduplicate_bismark Bismark (version 0.24.02) [49] and the cytosine report has been produced for each sample analysed, using the bismark_methylation_extractor function with the following parameters: –bedGraph –CX –buffer_size 10G –cytosine_report. To account for non-converted DNA, we applied a correction according to Catoni and Zabet [50]. Briefly, the number of methylated reads was decreased as: ${{m}^*} = \ {\mathrm{\lfloor }}\max ( {0,m - n(1 - c} )){\mathrm{\rfloor }}$, where m* is the adjusted number of methylated reads per cytosine position, *m* is the original number of methylated reads per cytosine position, *n* is the total number of reads per cytosine position, and *c* is the conversion rate.

To determine parent chromosomal recombination in the epiRILs, we call methylation in each gene using the analyseReadsInsideRegionsForCondition function in the DMRcaller package [36]. Then, we filtered genes that contain only CG methylation (mCG > 50%; CHG/CHH < 1%; min cytosine covered > 5), producing a list of 20 520 epigenetic markers. Then, using the same function, we calculated the CG methylation level of these markers for all epiRILs analysed (mCG_epi), relative to the methylation level calculated in the wild-type (mCG_WT) and *met1* mutant (mCG_met1) sample, with the following formula: (mCG_epi − mCG_met1)/(mCG_WT − mCG_met1). We then used the R *s*mooth.spline function (spar = 0.6) from the stats package to smooth data across chromosomes, and at any chromosomal location we assigned ‘Col-0-like’ methylation to smoothed values >0.8 and ‘met1-like’ methylation for values <0.2, leaving the rest classified as epi-heterozygous (lines still containing the two epialleles segregating) or undefined (for markers located at recombination points or in a gene-poor area of the genome), categories that were represented on average in <5% of each epiRIL genome.

### Identification of differentially methylated regions

To identify differentially methylated regions (DMRs), we used the DMRcaller package [35]. In particular, we selected the bins method with a bin size of 100 bp, used the statistical ‘score’ test (which is a z-test) and a *P*-value threshold of 0.01, and considered bins with at least four cytosines and at least four reads per cytosine. Moreover, based on our previous evaluation of MET1 mutants in Arabidopsis [35], for CG methylation, the minimum proportion difference was set to 0.4 (40% difference), while for CHG and CHH it was set to 0.1 (10% difference), in accordance with the lower magnitude of methylation in those contexts. Genes were split into three different groups, i.e. those overlapping only hypomethylated DMRs, those overlapping only hypermethylated DMRs, and those overlapping both hypomethylated and hypermethylated DMRs considering both a minimum overlap size between the gene and a DMR of 50 bp. This analysis was performed for all three methylation contexts and was repeated for promoters.

### Classification of genes by methylation

Genes were split into four categories, based on their methylation in Col-0: (i) genes with gbM (with CG methylation at least 10% and CHG and CHH methylation <5%); (ii) genes with TE-like methylation (with CHG or CHH methylation at least 5%); (iii) promoter corresponding to methylation in any context (with CG methylation at least 10% and/or CHG and CHH methylation of at least 5%) identified in the promoter region of a gene [1 kb upstream to 50 bp downstream of the transcription start site (TSS)]; and (iv) non-methylated genes (with CG methylation <10% and CHG and CHH methylation <5%). We considered the methylation state of the promoter independent of the methylation state of the gene, and thus we could have the case of the promoter being methylated in a non-CG context, but the gene body being methylated in a CG context only. We selected for our analysis genes where the number of total reads in Col-0 or MET1 mutants was at least 25. This was done for each biological replicate separately, and for both gene bodies and promoters. In MET1 mutants, genes hypermethylated in the CG context,were discarded.

### Corrected CV^2^

For the time series analysis, Figs 1 and 2 (and corresponding Supplementary Figs S1–S6), the CV is calculated once by pooling all measurements (all times and all replicates) for a given gene, while for analysis of the epigenetic mutants and epiRILs in Figs 3–5 (and corresponding Supplementary Figs S7–S15), the corrected CV^2^ is calculated at a single time point using multiple biological replicates only. In the case of the former, we measure the correlation between gbM and a combination of both intra- and inter-plant variability in gene expression, while, in the case of the latter, we measure the link between loss of gbM and an increase in inter-plant gene expression variability.

To adjust for the negative correlation observed between CV^2^ and mean expression level, we used a corrected CV with the approach previously described in [51]. Briefly, for each gene, we computed the log_2_ of the CV^2^ over the trend corresponding to the same expression level. The trend corresponding to the same expression level was computed using the glmgam.fit function. For this analysis, we selected only genes that have at least five transcripts per million.

### Gene expression comparison between Col-0 and MET1 mutants

Genes with significant expression change were defined here as those with an FDR <0.05, and log2 FC >1 or < −1. They were excluded from further analysis, in order to avoid bias caused by expression differences. The list of genes and the change in gene expression variability and methylation in the *met1-1* mutant are given in Supplementary Table S1, while those in the *met1-3* mutant are given in Supplementary Table S2.

For the epiRILs, we assigned each gene as being in either the Col-0 or *met1-3* epigenetic profile in each of the 10 epiRILs analysed, based on the genomic analysis described above (see also Supplementary Table S3). Genes for which an epigenetic profile was not clearly identified (because they were either close to a recombination point or in a epi-heterozygous area), genes that had mean FPKM (fragments per kilobase of transcript per million mapped reads) value <0.5 and genes that had more than one replicate with expression of 0 were removed from the analysis. We then selected the genes that are present in at least two epiRILs in the Col-0 epigenetic profile and in two epiRILs in the*met1-3* epigenetic profile, and grouped their expression levels based on the assigned genotype, considering them as ‘pseudo-replicates’, pseudo Col-0 or pseudo *met1-3*. Each epiRIL had two biological replicates and each gene can have a different number of pseudo-replicates varying between 4 and 16 since the epiRILs contain different recombination points between the Col-0 and *met1-3* chromosomes used in the original cross. We randomly selected three raw count values for Col-0 and three for *met1-3*, and used DESeq2 to call differential gene expression, an FDR threshold of 0.05, and no value for log2 FC. In our approach, epiRILs are treated as grouped pseudo-replicates and, to minimize the possibility that upstream effects do not lead to changes in gene expression, we selected no value for log2 FC. In addition, this was repeated 200 times and the genes that were not differentially expressed in any of the 200 iterations were selected. Genes that were not differentially expressed were taken forward and we computed the corrected CV^2^ (see ‘Corrected CV’ section) of the expression in pseudo Col-0 or pseudo *met1-3*, which further corrected for any effects of changes in mean levels of gene expression on variability in gene expression. We performed 200 iterations sampling three FPKM values for Col-0 or for *met1-3* and computed the corrected CV^2^. We then reported the mean corrected CV^2^ from the 200 iterations. Genes that display a difference in corrected CV^2^ between Col-0 and *met1-3* pseudo-replicates of at least 0.5 were considered to have increased/decreased variability in gene expression. The list of genes and the change in gene expression variability and methylation in epiRILs are given in Supplementary Table S4.

### Gene Ontology analysis

We used two external tools to perform Gene Ontology (GO) enrichment analysis: (i) Panther Over-representation Test [52] and (ii) DAVID [53]. Panther and DAVID analyses of the consensus gene set were conducted twice, using two backgrounds: first, the ‘all Arabidopsis genes’ background, built into both tools; and, second, the custom background, generated from all genes that were included into the Genomic Ranges object. All other Panther and DAVID analyses made use of the custom background, and no background was required for Wilcoxon analysis. The results of enrichment analysis were compared using q-value metrics. Go_enrich was corrected to match the output of Panther and DAVID manually, using the p.adjust function of the stats package (Supplementary Table S5).

### Antisense transcription analysis

We performed a similar analysis for our *met1-1* and *met1-3* mutants to that in Choi *et al.* [54]. In particular, we counted all genes that increase variability in gene expression and overlap with either an up-regulated or a down-regulated gene on the opposite strand, e.g. we selected genes with increased corrected CV^2^ on the positive strand that overlapped at least one up-regulated gene on the negative strand, and vice versa. We repeated the analysis for genes that decrease variability in gene expression and for genes that have an unchanged variability in gene expression.

## Results

### Gene expression variation in *A. thaliana*

To investigate variation in gene expression, we considered two publicly available time series expression datasets produced on leaf 7 of 5-week old Arabidopsis plants: (i) the well-watered samples of a progressive drought experiment (drought mock—discovery dataset); [28], which consisted of 14 daily time points; and (ii) the low-light-grown samples of a 6 h high-light exposure sampled at 30 min intervals (high light mock—validation dataset) [55]. Both datasets consisted of four biological replicates at each time point.

**Figure 1.**
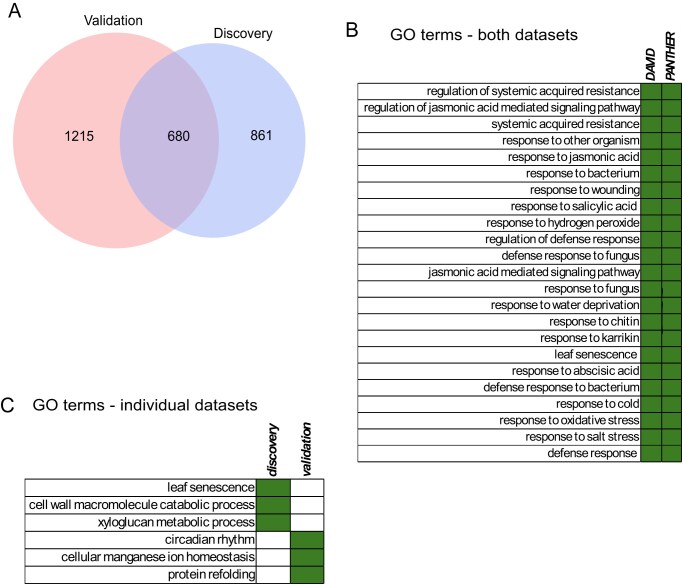
Genes with high gene expression variability in both datasets. (**A**) Venn diagram showing the overlap between genes with high gene expression variability in the discovery and validation datasets. (**B**) Significantly enriched GO terms (green squares) in genes that are variable in both datasets present in the output of two GO tools, DAVID and PANTHER. (**C**) Significantly enriched GO terms (green squares) of genes that are variable in either the discovery or validation dataset, encompassing genes in the overlap. Plots represent a manually selected subset of GO terms; full lists, including adjusted *P*-values and fold enrichment, can be found in Supplementary Table S5.

First, to compute variation in all biological replicates at all time points, we identified a set of probes present in both datasets, which resulted in a total of 19 239 genes for downstream analysis (Supplementary Fig. S1). Based on the outcomes of principal component analysis (PCA), we removed five discovery and two validation biological replicates from the downstream analysis due to their divergence from the remaining biological replicates (Supplementary Fig. S2A, B). The CV was selected as the metric of gene expression variability as previously described [56]. Based on the distribution of values across the two series (Supplementary Fig. S2C), 0.04 was chosen as the cut-off value, to categorize genes asstable or variable for further analysis. Based on this rule, 1542 genes in the discovery dataset and 1895 in the validation dataset were classed as variable. Of these, 680 were in common between both datasets, which is more than expected by chance (χ^2^ test *P*-value = 1.15 × 10^−6^), and we termed this the consensus list of variable genes (Fig. 1A).

GO analysis of the consensus list suggests that most of the genes respond to either an abiotic or a biotic stimulus or were involved in the regulation of stress responses (Fig. 1B). This suggests that gene expression variability may be involved in stress responses in plants. Interestingly, while validation-specific variable genes were enriched for circadian rhythm, the discovery-specific genes were enriched in leaf senescence and other processes linked to plant growth and development (Fig. 1C). This can be explained by the different times cales (hours versus days/weeks) of both datasets, with the discovery dataset including days/weeks as time points and the validation dataset including minutes/hours as time points.

### Gene body methylation is associated with stable gene expression

Next, we wanted to investigate if there is a link between variation or stability in gene expression and epigenetic signatures. Thus, we used publicly available bisulfite sequencing data of the *A. thaliana* Col-0 ecotype generated in 3-week-old leaves by Stroud *et al.* [33, 34]. The low-resolution profile analysis of Col-0 BS-seq data showed that the three biological replicates were largely similar, with the area of the centromere highly methylated compared with the chromosome arms (Supplementary Fig. S3A–C).

To investigate the link between DNA methylation and gene expression, we grouped genes into four classes: (i) gene body (gbM), corresponding to only CG methylation detected in gene bodies [from the TSS to the transcription end site (TES)] with CG methylation at least 10% and CHG and CHH methylation <5%; (ii) transposable-like, corresponding to methylation of at least 5% in CHG and CHH contexts found in gene bodies; (iii) promoter corresponding to methylation in any context (with CG methylation at least 10% and/or CHG and CHH methylation of at least 5%) identified in the promoter region of a gene (1 kb upstream to 50 bp downstream of the TSS); and (iv) non-methylated genes (with CG methylation <10% and CHG and CHH methylation <5%). We considered the methylation state of the promoter independent of the methylation state of the gene, and thus we could have the case of a promoter being methylated in a non-CG context, but the gene body being methylated in a CG context only. Note that we annotated every gene that displays methylation in a non-CG context above 5% as transposable-like methylation, similar to our previous approach [40].

**Figure 2.**
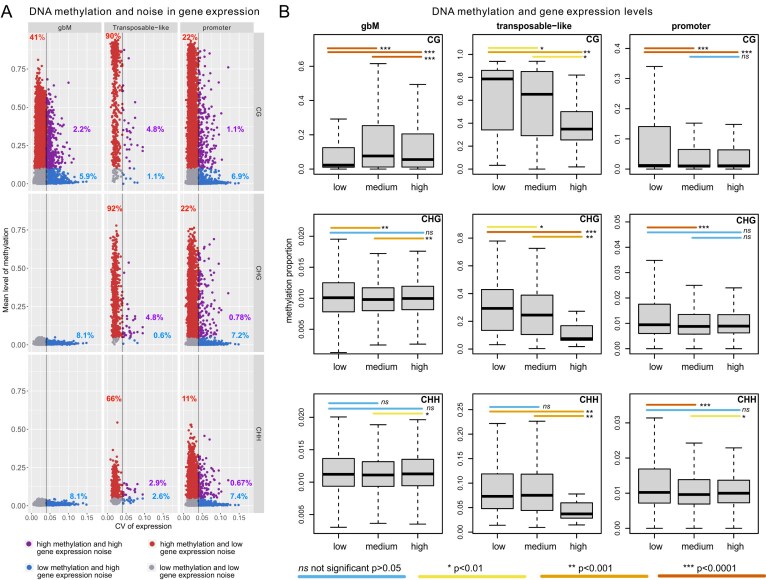
Relationship between methylation level, CV for gene expression, and level of gene expression in the discovery dataset. We considered the gene expression dataset for controls in Bechtold *et al.* [28]. (**A**) Link between methylation level and noise in gene expression. We considered separately the case of gbM, transposon-like methylation, and methylation at promoters. In addition, we consider context-specific methylation, by splitting the methylation in CG, CHG, and CHH contexts. Red points represent genes with a high level of methylation and low noise in gene expression, blue points represent genes with a low level of methylation and high noise in gene expression, magenta points represent genes with a high level of both methylation and noise in gene expression, while grey points represent genes with both a low level of methylation and noise in gene expression. (**B**) Statistical analysis of the relationship between gene expression magnitude and methylation proportion in different methylation contexts and methylation types. The plots are grouped by methylation type: gbM genes (left-hand column), genes with TE-like methylation (middle column), and promoter methylation (right-hand column), divided into different methylation contexts, CG, CHG, and CHH. In addition, genes are grouped into three categories based on their expression level: (i) low expression with values <8.4; (ii) medium expression with values between 8.4 and 12.05; and (iii) high expression with values >12.05. Only genes falling into the same category in both the discovery and validation datasets were analysed. *P*-values were calculated using Wilcoxon rank-sum test. The colour of the lines between two boxes indicates the statistical relationship between the two distributions as calculated by Wilcoxon rank-sum test, as shown on the figure.

A high level of methylation corresponds to lower variation in gene expression (41%) (Fig. 2A, red dots). In contrast, a lower level of methylation corresponds to higher variation of gene expression (5.9%) (Fig. 2A, blue dots), with relatively few genes (2.2%) displaying both high methylation and a high level of variation (purple dots). This is particularly true for gbM in the CG context and promoter methylation in all contexts, and these results are reproducible in the validation dataset (Supplementary Fig. S4A). Furthermore, the differences in methylation between stable and variable genes in the different groups are statistically significant (except for non-CG methylation at genes with gbM, Supplementary Fig. S5). The statistical analysis further confirms that the most significant difference is observed for gbM (Supplementary Fig. S5).

Considering that DNA methylation is associated with transcriptional silencing, one possibility is that methylated genes are expressed at a lower level, and low expression could explain the lower variability. To explore this possibility, we evaluated the correlation between methylation levels and levels of expression. For genes with TE-like methylation, this was indeed the case, with highly expressed genes having low or no methylation (Fig. 2B; Supplementary Fig. S6). Nevertheless, for gbM and promoter methylation, high levels of methylation were not associated with a low level of expression (Fig. 2B; Supplementary Fig. S6). In fact, a medium and high level of gbM correspond to a higher level of gene expression compared with low levels of gbM (Fig. 2B). Again, these results are reproducible in the validation dataset, confirming that they are not affected by the dataset used in our analysis (Supplementary Fig. S4B). Given that the validation and discovery datasets are measured at different time scales (hours versus weeks), this suggests that the results are independent of effects induced by developmental or circadian regulation.

### Loss of gene body methylation in MET1 mutants increases gene expression variability

So far, our results have shown a correlation between gbM and stability of gene expression. To further investigate if there is a functional link between the two, we considered data in *met1* mutants that are hypomethylated in CG methylation. In particular, we used methylation and expression datasets from two *met1* mutants: (i) *met1-1* that results in a loss of approximately three-quarters of CG methylation and (ii) *met1-3* that results in a complete loss of CG methylation (Supplementary Fig. S7). The CG methylation that is retained in the*met1-1* mutant is mainly located in promoters, TEs, and non-CG methylated regions, but almost all gbM is lost (Supplementary Fig. S8A) [35]. This contrasts with the *met1-3* mutant where virtually all CG methylation is lost, including in promoters, gene bodies, and TEs.

**Figure 3.**
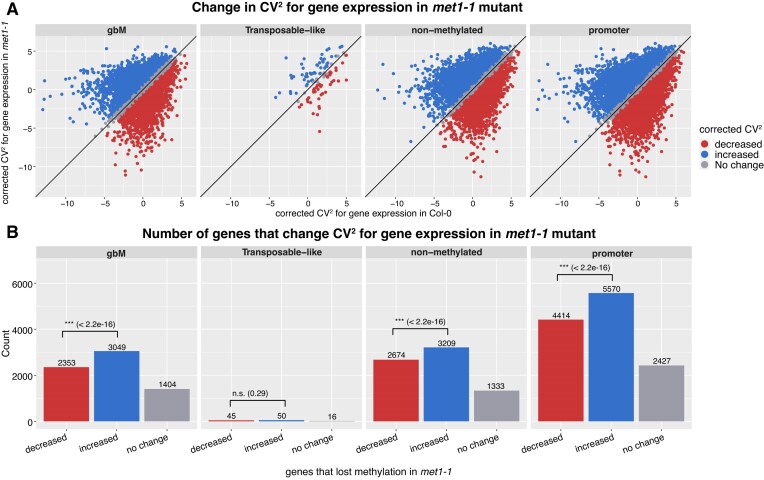
Changes in noise in gene expression in the *met1-1* mutant. (**A**) Comparison between the corrected CV^2^ in Col-0 and *met1-1* plants; data from [35]. We considered in this analysis only genes without a significant change in gene expression between Col-0 and the mutant but which show loss of DNA methylation (overlap with a hypomethylated DMR in *met1-1*). Genes are grouped based on their CV change: (blue) increased for genes with corrected CV^2^ FC >0.5, (red) decreased for genes with corrected CV^2^ FC < –0.5, and (grey) no change for those in between. (**B**) The number of genes in each group shown in (A).

**Figure 4.**
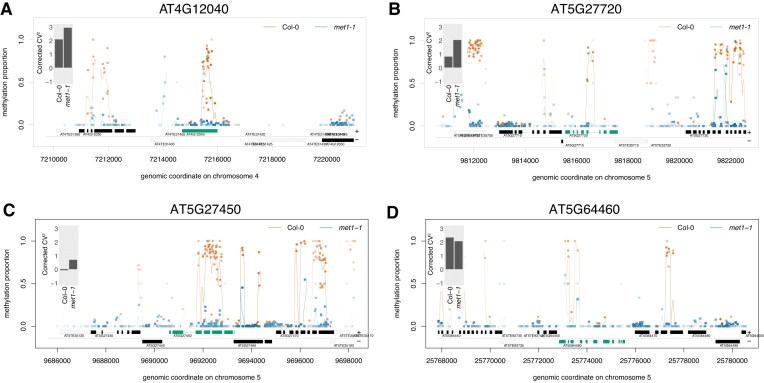
Loss of gbM results in an increase in noise in gene expression. We considered four examples: (**A**) AT4G12040, (**B**) AT5G27720, (**C**) AT5G27450, and (**D**) AT5G64460. The red colour represents methylation in *the met1-1* mutant and the blue colour in Col-0 plants. The gene of interest is highlighted by marking the exons in green. The inset represents the corrected CV^2^ levels for the corresponding gene. In (A–C), the complete removal of gbM results in a large increase in gene expression noise. In (D), the retention of approximately half of gbM results in maintenance of the same level of gene expression noise.

In both *met1-1* and *met1-3* mutants, there is a subset of genes that are up-regulated compared with Col-0, but also a large subset of genes that maintain the same level of expression (Supplementary Fig. S8B, C). To investigate the effect of methylation loss on gene expression variability, we considered only genes (and promoters) that lost CG methylation but maintained the same level of gene expression compared with Col-0. We applied a correction to the CV (see the Materials and methods and Supplementary Fig. S9) using the approach previously described [51]. This ensures that the effects on variability of gene expression are not caused by the change in levels of gene expression, but only by the loss of CG methylation. Independent of where the methylation is located (gene bodies or promoters) or the type of methylation (transposon like), a significantly higher number of genes show increased variability in expression in the *met1-1* mutant (Fig. 3). This is statistically significantly higher than the number of genes with decreased variability in gene expression. One of the largest effects is observed for gbM, where >3000 genes increase their variability in gene expression upon loss of CG methylation compared with >2300 genes that display a decrease in variability in gene expression. Figure 4A–C shows an example of three genes (AT4G12040, AT5G27720, and AT5G27450) that completely lose gbM in *the met1-1* mutant and show an increase in the gene expression variability. In contrast, AT5G64460 retains approximately half of gbM in *the met1-1* mutant and there is a negligible corresponding change in gene expression variability (Fig. 4D).

In contrast to what we have seen in the*met1-1* mutant, in *met1-3* we observed a lower number of genes that increase their gene expression variability, indicating that although both mutants affect the variability in expression of many genes, only in *met1-1* does this result in an overall increase of variation in expression (Supplementary Fig. S10A, B). There are several possibilities that could explain these results. First, the set of genes that lost DNA methylation in *met1-1* could be more prone to display an increase in noise upon loss of DNA methylation. However, most genes that have lost gbM are common to both the *met1-3* and *met1-1* mutant. This suggests that the effect on gene expression variation observed in *met1-1* is not driven by a specific set of genes with altered methylation specifically in the *met1-1* mutant.

Secondly, it is possible that the* met1-3* mutant, being a complete lack of function allele, accumulates larger epigenetic and transcriptomic changes and, consequently, more indirect effects occur. To investigate how many of these changes in variability in gene expression in *met1-3* are also found in *met1-1*, we investigated the overlap of genes in the two mutants that display either an increase or a decrease in variability of gene expression (Supplementary Fig. S11). There is a large overlap between genes that lose gbM in both mutants and increase variability of expression (1357 genes), which is the largest overlap between the two mutants for any of the categories of genes.

### Gene expression variability in epigenetic recombinant inbred lines

**Figure 5.**
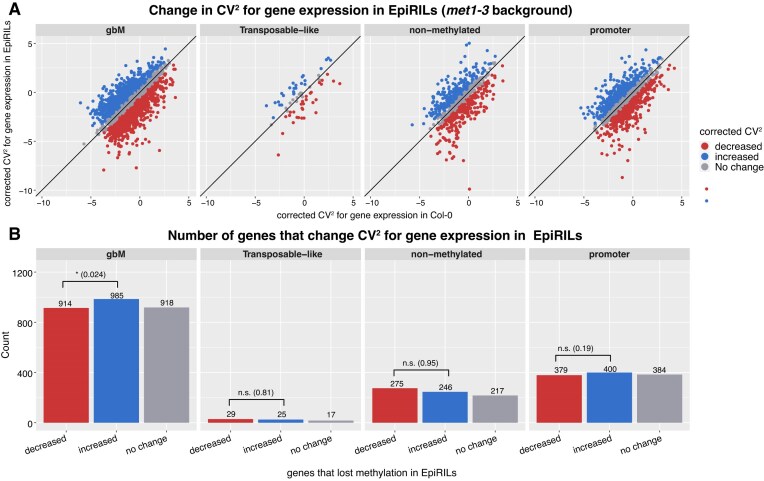
Changes in noise in gene expression in 10 epiRILs. (**A**) Comparison between the corrected CV^2^ in genes in the Col-0 background and those in the *met1-3* background. We considered in this analysis only genes without a significant change in gene expression when in Col-0 and the mutant backgrounds but appearing in the *met1-3* background in at least two epiRILs. Genes are grouped based on their change in CV: (blue) increased for genes with corrected CV^2^ FC >0.5, (red) decreased for genes with corrected CV^2^ FC < –0.5, and (grey) no change for those in between. (**B**) Number of non-differentially expressed genes with loss of DNA methylation between Col-0 and *met1-3* backgrounds in the different categories shown in (A).

Furthermore, to investigate if the asymmetric change in CV observed in *met1-1* was masked in the* met1-3* background due to the high number of genes that change expression, we took advantage of epiRILs generated by a cross between Col-0 and *met1-3* plants, and propagated by single seed descent [57, 58]. Each epiRIL plant displays parts of the genome inherited from a Col-0 epigenetic state and parts from the genome in the *met1-3* epigenetic state, but they all contain a wild-type allele of the MET1 gene. By analysing their WGBS dataset, we were able to annotate in each line the parts of the genome that are Col-0 or *met1-3*. We then performed RNA-seq for 10 epiRILs in two biological replicates. In each epiRIL, genes were labelled as either Col-0 or *met1-3*, depending on whether the genomic region where the gene was located was in the Col-0 or *met1-3* background. We selected the genes that appeared at least twice in the Col-0 background and twice in *the met1-3* background in the 10 epiRILs and grouped their expression levels based on the background they come from. This resulted in generating pseudo Col-0 and pseudo *met1-3* replicates for each gene (see the Materials and methods). We selected the genes that maintained their expression level independent of whether they were in the Col-0 or *met1-3* background, estimated the corrected CV^2^ in gene expression among the pseudo-replicates in Col-0 and *met1-3*, and corrected the CV (Supplementary Fig. S9) using the approach previously described [51]. Our results showed that 985 genes with gbM increased variability in expression when in *the met1-3* background compared with Col-0, and this is higher than the number of genes that decreased variability (914), with the difference being statistically significant; see Fig. 5. This indicates that the larger number of genes that decrease gene expression variability in *met1-3* could be caused, at least partly, by the high number of genes that change expression and the corresponding indirect effects.

### Asymmetry of CG methylation in the *met1-1* mutant is linked with variability in gene expression

Since approximately a quarter of CG methylation is retained in *met1-1*, another possibility is that methylation is lost preferentially at genes that increase their variability in expression, which appears not to be the case (Supplementary Fig. S12A). Alternatively, the *met1-1*-specific increase in variation could reflect variation at the level of the DNA molecules analysed in the sampled tissue, at the level of alleles or the single-cell epigenetic landscape. This could be explained by a partial or incomplete activity of MET1, which is consistent with the lower stability of MET1 protein observed in this mutant [40].

MET1 methyltransferase is responsible for maintaining methylation at symmetric CG sites, and thus the observation of asymmetry in CG could be associated with incomplete MET1 activity. Therefore, we looked specifically at whether methylation could be maintained at both cytosines in a CG site or if one cytosine loses methylation while the other cytosine maintains it. Our results show that at genes that increase variability in expression in *the met1-1* mutant, there is a significant decrease in methylation symmetry at CG sites that are still methylated (Supplementary Fig. S12B). Nevertheless, this asymmetry in methylation at CG sites is also observed in genes that display a lower variability in gene expression, suggesting that asymmetry of DNA methylation alone cannot explain the buffering in noise in gene expression observed in *the met1-1* mutant.

### Decreased variability of expression in the *met1-3* mutant correlates with a reduced acquisition of non-CG methylation at gene bodies of a subset of genes

From the transcription data we have analysed, and from previous work, we observed an increase in non-CG methylation occurring in the *met1-3* mutant, which is likely to be the consequence of incorrect splicing of the histone demethylase IBM1 responsible for removing non-CG methylation in gene bodies [59, 60, 61]. Our results confirm that there is a relatively large increase of non-CG methylation (between 5% and 10%) in gene bodies and promoters in *met1-3*, but not in *met1-1* (Supplementary Fig. S13). Interestingly, we observed that in *met1-3*, non-CG (only CHH) hypermethylation is slightly more pronounced in gene bodies of genes with a decreased variability in gene expression than in those of genes with an increased variability in gene expression (Supplementary Fig. S13A, B), while this is not evident in the wild type or the *met1-1* mutant (Supplementary Fig. S13E, F). This effect is also absent in promoters, which display a similar increase in non-CG methylation irrespective of the change in variability of gene expression (Supplementary Fig. S13C, D). It is noteworthy that this low but significant accumulation of non-CG methylation at gene bodies does not lead to changes in gene expression at the genes considered in our analysis, i.e. we only selected genes that are not differentially expressed in *met1-3* for this analysis (Supplementary Fig. S8C).

Considering that repeated DNA has been associated with the ability to attract methylation [40], we investigated the presence of repetitive elements near (within 10 kb) genes that increase or decrease variability of expression in *the met1-3* mutant. We found an average of four annotated TEs in the proximity of genes of all groups (Supplementary Fig. S14A); however, we observed that there is preferential association of certain families near genes that decrease variability of expression (Supplementary Fig. S14B), including *ATMU* (6 and 9), *ATCOPIA* (50, 11, 22, 23, 34, 44, and 93), and *VANDAL* (9, 20, 7, and 1N1). Furthermore, other TE families are preferentially located near genes that increase variability of expression (Supplementary Fig. S14C), including *COPIA* (19, 26, 60, and 72) and *VANDA*L (10 and 15). These results suggest that CHH methylation accumulates in the *met1-3* mutant at a diverse subset of specific TE families, which could partially compensate the variability of expression gained by the loss of CG methylation in bodies. Therefore, the effect of increased variability in expression found in the *met1-1* mutant, at least for some genes, could be buffered in *met1-3* by the deposition of non-CG methylation. While our results demonstrate a relationship between DNA methylation and variability of gene expression, it is however likely that multiple mechanisms are involved in controlling the variability of expression, which could be directly or indirectly linked to epigenetic regulation.

### The reduction in gene expression variability in *met1-3* can be linked with increased antisense transcription but not with dynamic gene body methylation

One possible explanation for the increase in variability in gene expression is that loss of DNA methylation leads to an increase in antisense transcription, which in turn increases the variability in transcription. Indeed, a *met1* mutant (*met1-6*) was recently shown to display changes in antisense transcription [54]. We performed a similar analysis for our *met1-1* mutant and found only 14 genes with up-regulated antisense transcripts that have lost gbM in *met1-1* and where the gene is differentially expressed (Supplementary Fig. S15A). We also investigated the *met1-3* mutant and found more genes that have lost gbM and are not differentially expressed with up-regulated antisense transcripts (204). Interestingly, we observed that loss of antisense transcription in the *met1-3* mutant corresponds to an increase in gene expression variability, while gain of antisense transcription corresponds to a decrease in gene expression variability (Supplementary Fig. S15B). Nevertheless, this is the case for less than a fifth of the genes that show increased or decreased gene expression variability. Overall, antisense transcription might be linked with reduced variability in gene expression at a small but significant number of genes.

In *A. thaliana*, another study identified that gbM can be further classified into stable and dynamic, with the latter showing higher levels of gene expression variability during development and in diverse physiological stress conditions [62]. We investigated whether there are more genes with dynamic gbM in the set of genes that increase variability in *met1-1* or *met1-3* mutants, but our results showed that this is not the case (Supplementary Fig. S15C, D). Dynamic gbM has been linked with variability across development and physiological stress conditions, and the fact that our *met1* mutants were grown in constant conditions could explain the low overlap between genes with dynamic gbM and genes with increased variability in the*met1-1* mutant.

## Discussion

Noise is hardwired in biochemical systems, either as an intrinsic component from biochemical reactions or as extrinsic factor that is propagated through regulatory pathways [63–65]. Previous work has identified some potential sources of variation in gene expression between isogenic populations, but the finer details and the mechanism responsible for regulation of transcription variation are currently not fully understood. Our previous work showed that a reduction in noise in gene expression without slowing down the response time of genes can be achieved by: (i) increasing the metabolic cost associated with the gene [66]; (ii) having a negative feedback loop (the gene encodes a TF that represses its own expression) [67]; or (iii) the TF that regulates the activity of the gene performing facilitated diffusion (a combination of a 1D scan and 3D diffusion) [68].

Here we characterized genes that display noise in gene expression in a single leaf of the model plant *A. thaliana*. Our results show that in two different time series datasets consisting of 13 evenly spaced time points collected across different time scales, the genes displaying high variability in expression are enriched in those related to stress response. This is consistent with previous research, which has identified that genes responsible for stress response are highly variable in yeast [69] and in Arabidopsis, across a 24 h diurnal period, with different classes observed between the day and night-time [2]. In mangroves, it has also been shown that transgenerational inheritance of acquired gbM helps to improve robustness to environmental stress, by enabling long-term adaptation to stress, which is important when there is reduced genetic diversity in the population [70]. Both our datasets contained samples taken exclusively during the light period, but the discovery dataset was collected once a day over a 14 day period while the validation dataset was sampled every 30 min over a single 6 h light period. Interestingly, this difference in temporal scale between the discovery and validation datasets is reflected in GO terms that are associated with circadian rhythm and photosynthetic activity for the validation dataset, and processes involved in plant growth and development for the discovery dataset (Fig. 1C). The variable genes common in both datasets, however, are clearly enriched for abiotic and biotic stress response GO terms (Fig. 1B), which suggests that the link between gene expression variability and stress response is independent of time scales and the developmental stage of the rosette leaf. It has been suggested, however, that developmental stochasticity is caused by noisy gene expression, which may vary between tissues and/or cell types [7]. Both datasets were taken from the fully expanded rosette leaf 7, comprising a number of tissues and cell types, representing transcriptional heterogeneity in heterogenous cell types, and therefore may have a less significant effect on mean expression levels of the entire tissue. A potential concern is whether developmental changes in methylation during rosette maturation could confound the observed association between gbM and gene expression variability. However, there is little evidence in the literature of extensive developmental methylation changes in mature rosettes [71], with the majority of studies focusing on methylation changes during germination and seedling development [72, 73]. Furthermore, the differences in methylation between the wild type and *met1* mutants will be significantly greater than any difference that may occur in mature rosettes.

DNA methylation has been proposed to be involved in repression of gene transcription. In plants, while this seems to be the case at genes with transposon-like methylation (having both CG and non-CG methylation), it is not the case for genes having only gbM (DNA methylation only in the CG context located in gene bodies), which display medium and high expression levels [74]. Given the metabolic cost associated with establishment and maintenance of DNA methylation, this raises the question of what the functional role of DNA methylation in gene bodies is. In this study, we performed a systematic analysis and found a link between DNA methylation in gene bodies and variability in gene expression. In particular, we found that genes with gbM displayed low noise in gene expression, while genes lacking gbM displayed higher noise in gene expression. This link is not restricted to gbM, but is also present at promoters, where methylated promoters displayed stable gene expression while unmethylated promoters were affected by noise in gene expression. We identified this association using bulk expression datasets in both the 6 h and 14 day datasets. Recently, another study using single-cell RNA sequencing (scRNA-seq) datasets identified a similar anti-correlation between gbM and noise in gene expression [25].

One possibility is that there is only a correlation between gbM and noise in gene expression and that there is no functional relationship between the two. To further dissect this link between DNA methylation and noise in gene expression, we analysed expression and methylation datasets in two MET1 mutants, *met1-1* and *met1-3*. In particular, *met1-1* results in loss of approximately three-quarters of CG methylation genome wide, while *met1-3* results in complete loss of CG methylation [33, 35].

To ensure that differences in sequencing depth between samples did not confound our gene expression variability estimates, we down-sampled all libraries to 9 million reads. Genes with low expression levels can lose a portion of their signal when down-sampled, making their expression estimates potentially noisier.

Our results show that the loss of gbM in *met1-1* resulted in an increase in variability of gene expression (Figs 3 and 4). This is statistically significant, and it is true when we selected only genes that maintain the same level of gene expression and corrected the CV to account for changes in gene expression, thus excluding that this increase in gene expression variability is linked to genes changing expression levels. Furthermore, we also found that there are more genes that display higher noise even upon loss of CG methylation at promoters. Interestingly, this was not the case with the *met1-3* mutant, where there is no predominant increase in noise in gene expression (Supplementary Fig. S10).

Due to the large number of genes that change expression in *met1-3*, it is possible that there are many indirect effects where upstream regulators are changing expression in *met1-3* which lead to changes of variability in gene expression for some downstream genes. If this were to contribute to the higher number of genes that decrease variability in gene expression in the *met1-3* mutant, then we would expect that epiRILs, where only a subset of the genome is in the *met1-3* background, would display a higher number of genes that increase variability in gene expression. This is indeed the case and, while the difference between genes that increase variability in gene expression and those that decrease it is small, it is statistically significant (Fig. 5).

In addition, it seems that low but significant accumulation of non-CG methylation in gene bodies can explain the decrease in transcription variability at genes in *met1-3* (Supplementary Fig. S13). Overall, we have a model where CG methylation buffers noise in gene expression and loss of CG methylation corresponds to an increase in transcription variability, but this can be compensated by an increase in non-CG methylation in gene bodies of certain genes or by indirect effects of changes in gene expression of upstream regulators.

In *met1-1*, there is loss of CG methylation mainly in gene bodies, but in *met1-3* there is loss of CG methylation genome wide, including repeats and TEs. One possibility is that the loss of CG methylation at TEs can increase non-CG methylation at gene bodies, by RdDM mechanisms [75]. Our results showed that the promoters of both genes that display an increase or a decrease in variability in *met1-3* have a similar number of proximal TEs, but different families of TEs are present at promoters of genes that decrease variability of expression compared with genes that increase variability of expression in *met1-3*.

It is worth noting that our analysis cannot distinguish between the intrinsic and the extrinsic components of noise, where stochasticity in RNA polymerase II (Pol II) binding and the transcription rate would represent the intrinsic component and stochasticity in TFs to control gene expression would represent the extrinsic component. DNA methylation at promoters would impact both binding of TFs and Pol II, and thus could contribute to both the intrinsic and the extrinsic component of the noise. In gene bodies, DNA methylation could impact both Pol II elongation and binding of TFs to intronic enhancers, and thus would also contribute to both the intrinsic and extrinsic components. While intronic enhancers in Arabidopsis have been considered not to be as prevalent as in animals, recent reports identified ~1000 putative intronic enhancers and validated a small subset of those [76].

Noise can be evaluated under both constant [26] and changing conditions [27]. In the MET1 mutants and epiRILs, we estimated the variability in gene expression under constant conditions, while in the case of the control time courses, the variability in gene expression was estimated under changing conditions, with time thus representing a mixture of temporal dynamics and inter-plant variability.

For the former, intrinsic noise is the main contributor to the overall observed noise in gene expression, while, for the latter, extrinsic noise would also make a significant contribution.

In human immune cells, previous work showed that DNA methylation variability in promoters, but not in gene bodies, is positively correlated with variability in gene expression [77]. A similar link between DNA methylation variability in 5′-untranslated regions (UTRs) and variability in gene expression was found recently in a study in human whole blood [78]. Nevertheless, another study showed that, in human immune cells, DNA methylation had a smaller contribution to gene expression variability compared with genetic variation or histone modifications, and significantly affects only a small number of genes (<250) [79]. This suggests that DNA methylation would control gene expression variability in humans as well, but at a much lower scale and potentially in promoters and *cis*-regulatory regions rather than in gene bodies.

Finally, it is worth noting that here we focused on gene transcription, which does not always accurately predict concentration of protein [80]. As such, it is possible that post-transcriptional mechanisms would moderate the effects of variation in mRNA concentrations.

## Supplementary Material

gkag127_Supplemental_Files

## Data Availability

The epiRIL WGBS and RNA-seq datasets from this study have been submitted to the NCBI Gene Expression Omnibus (GEO; http://www.ncbi.nlm.nih.gov/geo/) under accession numbers GSE255301 (RNA-seq) and GSE255302 (WGBS). The RNA-seq datasets for Col-0, met1-1, and met1-3 have been submitted to the European Nucleotide Archive (ENA; https://www.ebi.ac.uk/ena/) under accession E-MTAB-16182. All scripts used for pre-processing and further analysis were deposited on Figshare and can be accessed at https://figshare.com/s/9ee41d4c8862bea2d96c.
