# Supplementary material for: Gene body methylation buffers noise in gene expression in plants
Source: Nucleic Acids Res. 2026 Feb 17;54(4):gkag127. doi: 10.1093/nar/gkag127 (PMC12910109; doi:10.1093/nar/gkag127)
Supplement: gkag127_Supplemental_Files [file gkag127_supplemental_files.zip › gbM_expression_supp_R3.pdf]

Supplementary Material to:

Gene body methylation buffers noise in gene expression in plants

Jakub Zastąpiło[1], Robyn A Emmerson[2], Liudmila A Mikheeva[3], Marco Catoni[2,*], Ulrike Bechtold[1,4,*] and Nicolae Radu Zabet[1,5,6,*]

Supplementary Tables

Supplementary Table S1. List of genes and the change in gene expression variability and methylation in *met1-1* mutant.

Supplementary Table S2. List of genes and the change in gene expression variability and methylation in *met1-3* mutant.

Supplementary Table S3. The epigenetic state and gene expression levels of all genes in epiRIL lines.

Supplementary Table S4. List of genes and the change in gene expression variability and methylation in epiRILs.

Supplementary Table S5: List of significant enriched GO terms in variable genes: (i) genes common in the discovery and validation datasets, and (ii) all variable genes in either the discovery or validation datasets.

Supplemetary Figure

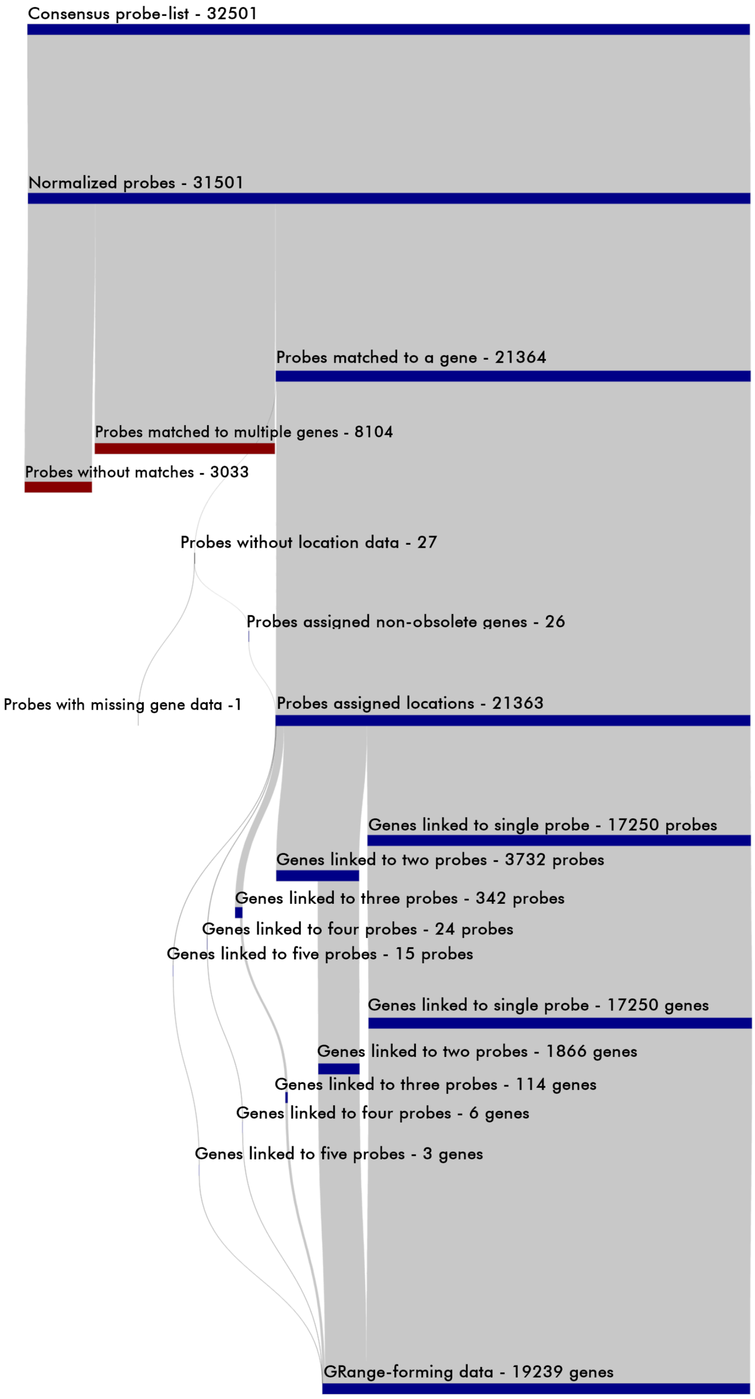

Supplementary Figure S1. *Microarray expression data processing.* Sankey diagram representing the steps taken to process the merged discovery and validation microarray data. Bars in red represent rejected probes, bars in blue represent probes or genes passed on to further analysis. Each flow represents the number of genes/ probes passed for the next analysis step.

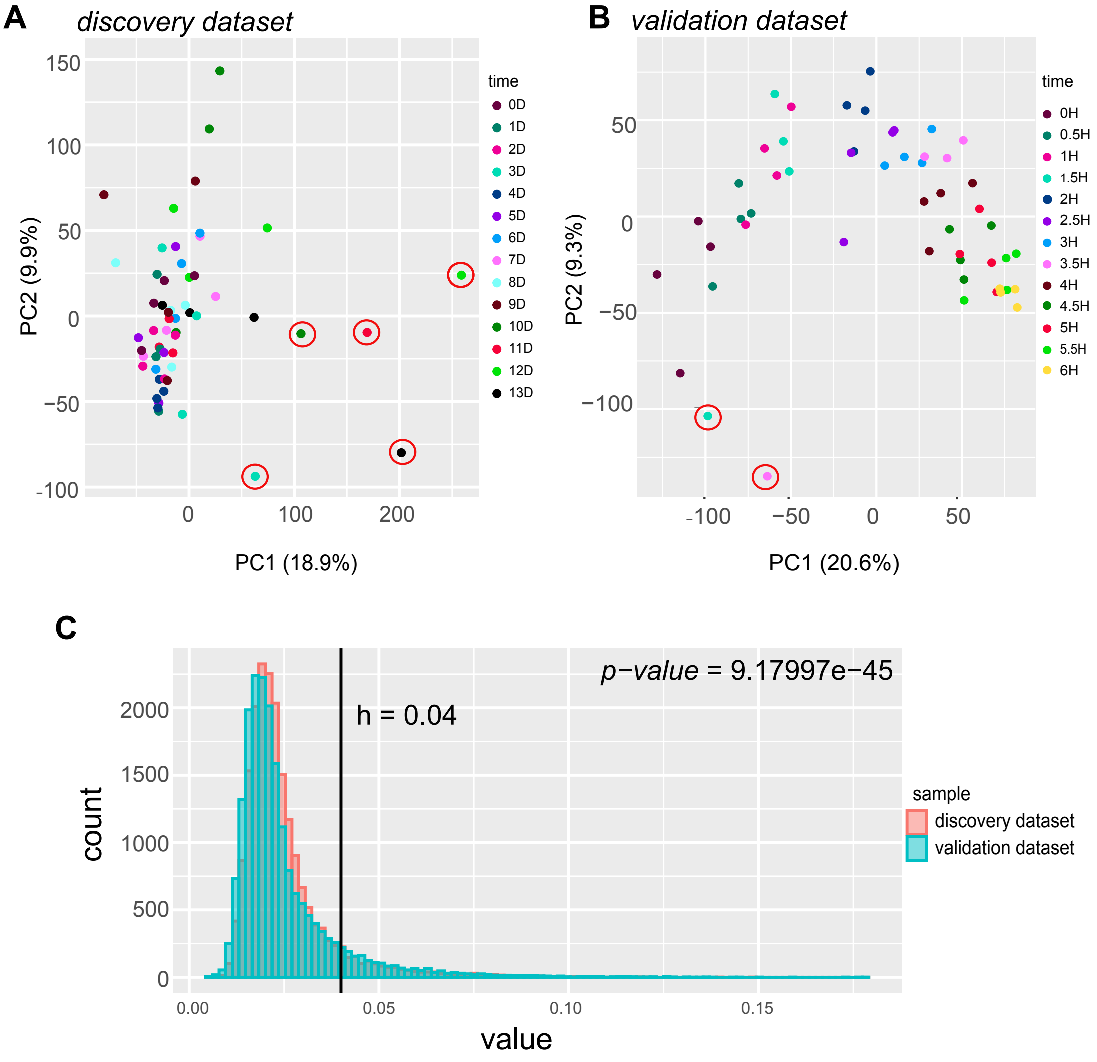

Supplementary Figure S2. *Principal Component Analysis (PCA) and analysis of variance. (A)* Discovery dataset PCA. Each datapoint corresponds to a single biological replicate and biological replicates are grouped by colour and are labelled according to their time point in hours. Datapoints highlighted in red circles were removed from further analysis. *(B)* Validation dataset PCA. Each datapoint corresponds to a single biological replicate and biological replicates are grouped by colour and are labelled according to their time point in hours. Datapoints highlighted in red circles were removed from further analysis *(C)* Coefficient of variation (CV) for the discovery (blue) and validation (red) datasets. The vertical line represents a cut-off point set at 0.04 based on the distribution of the values across the two-time series. Values above this cut-off point were classified as variable.

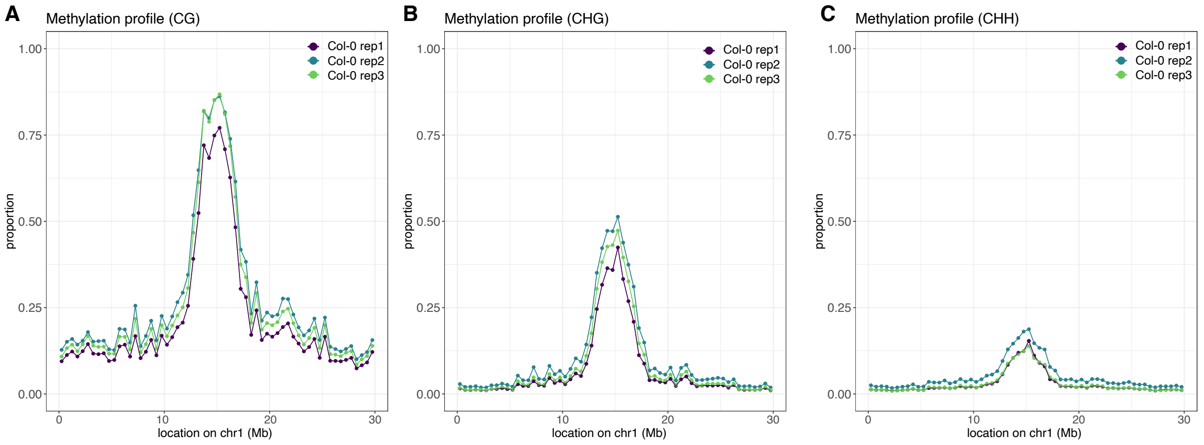

Supplementary Figure S3. *Low resolution (500 Kb) methylation profiles of chromosome 1 in Col-0 (A)* CG, *(B)* CHG and *(C)* CHH context.

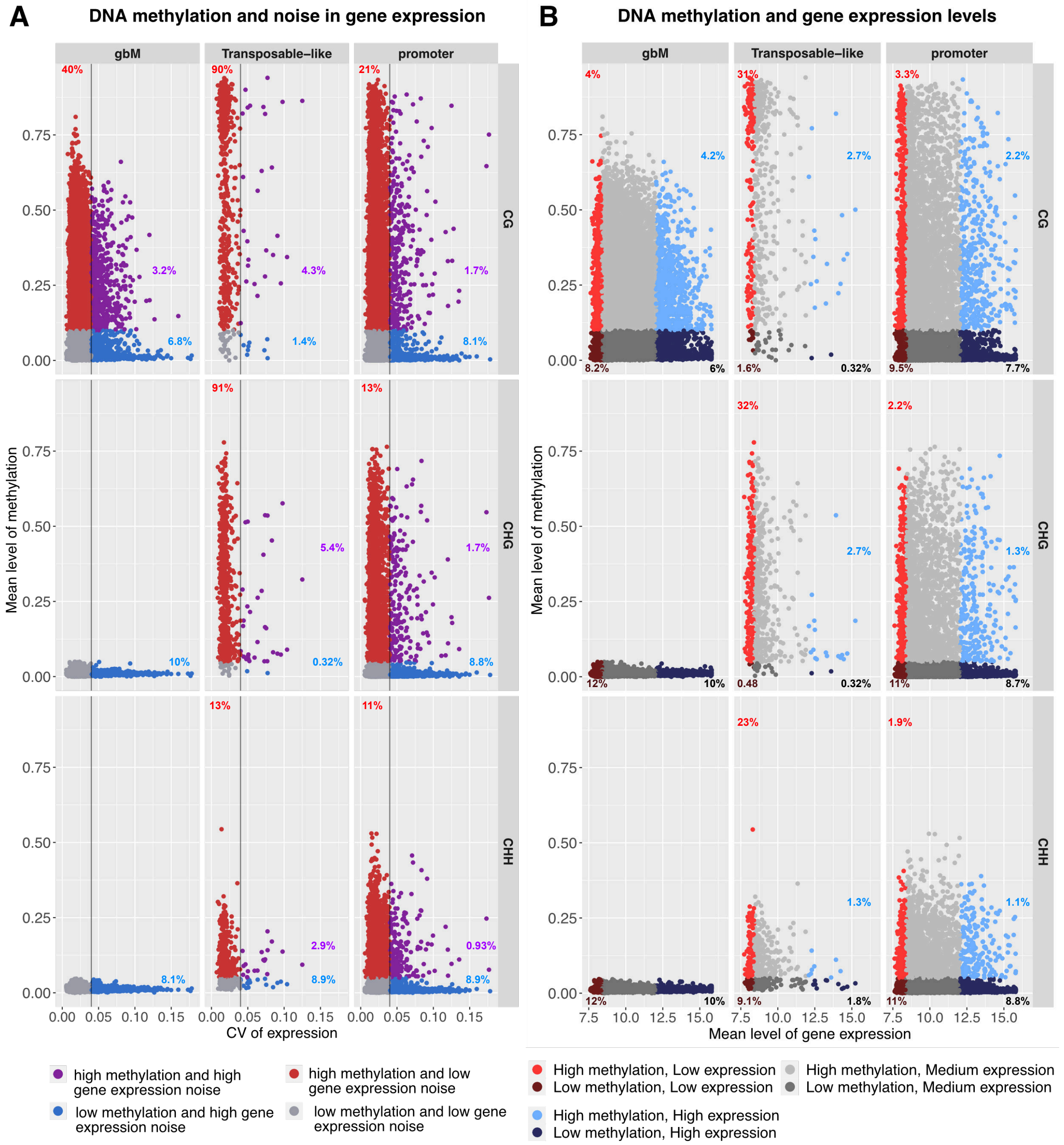

Supplementary Figure S4. *Relationship between methylation level, coefficient of variation for gene expression and level of gene expression in the validation dataset.* Same as Figure 2 in main manuscript but using the validation datasets. *(A)* Link between methylation level and noise in gene expression. *(B)* Link between methylation level and level of gene expression. (*A*-*B*) We considered separately the case of gene body methylation (gbM), transposon like methylation and methylation at promoters. In addition, we consider context specific methylation, by splitting the methylation in CG, CHG and CHH contexts. Red points represent genes with high level of methylation and low noise in gene expression, blue points represent genes with low level of methylation and high noise in gene expression, magenta points represent genes with high level of both methylation and noise in gene expression, while grey points represent genes with both low level of methylation and noise in gene expression.

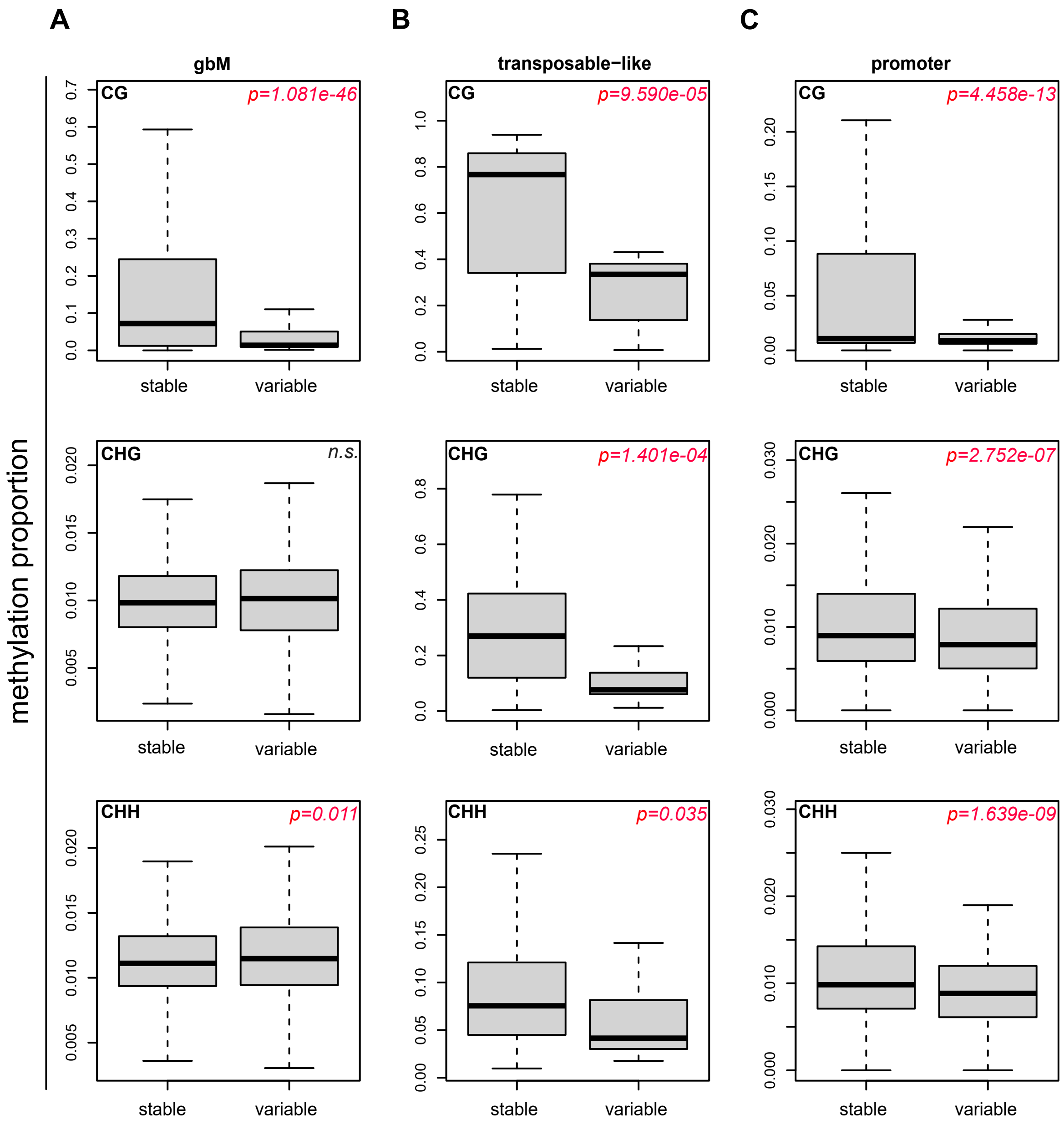

Supplementary Figure S5. *Relationship between CV of expression and methylation proportion in different methylation contexts using the validation dataset.* Genes were divided into two categories based on the covariance of expression: variable (CV > 0.04) and stable (CV < 0.04). We considered separately the case of: (A) gene body methylation (gbM), (B) transposable-like elements, and (C) promoters. P-values were calculated using the Wilcoxon rank-sum test. Significant p-values are provided, n.s. – not significant. This plot uses the validation datasets.

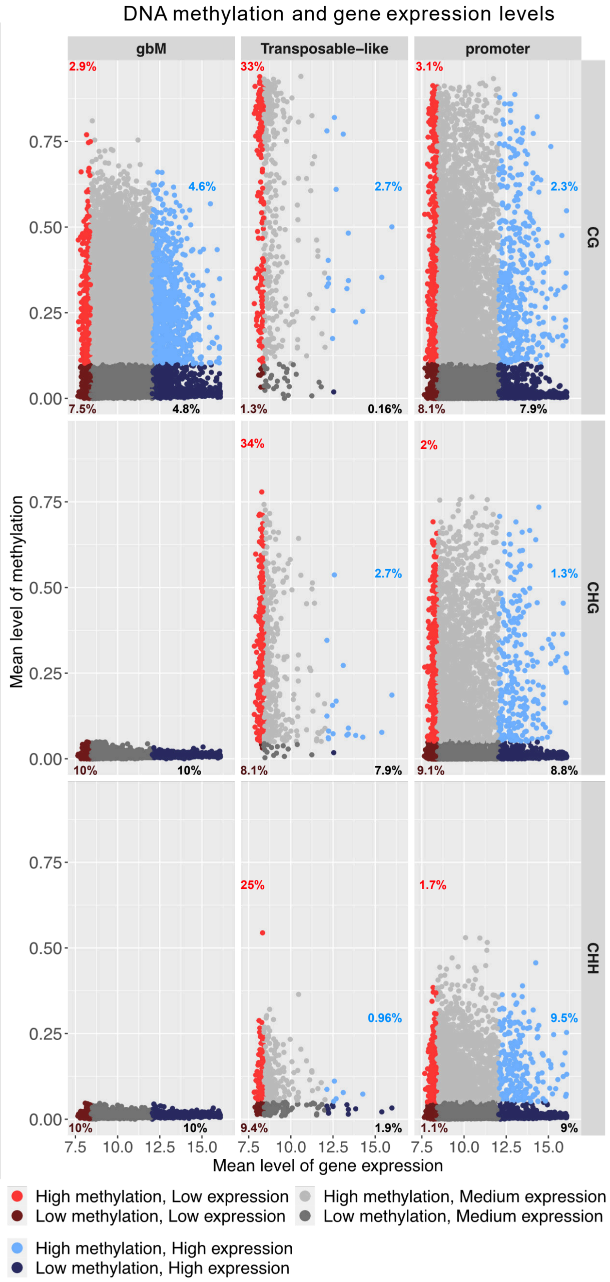

Supplementary Figure S6. *Link between methylation level and level of gene expression in the discovery dataset*. We considered separately the case of gene body methylation (gbM), transposon like methylation and methylation at promoters. In addition, we consider context specific methylation, by splitting the methylation in CG, CHG and CHH contexts. Red points represent genes with high level of methylation and low noise in gene expression, blue points represent genes with low level of methylation and high noise in gene expression, magenta points represent genes with high level of both methylation and noise in gene expression, while grey points represent genes with both low level of methylation and noise in gene expression.

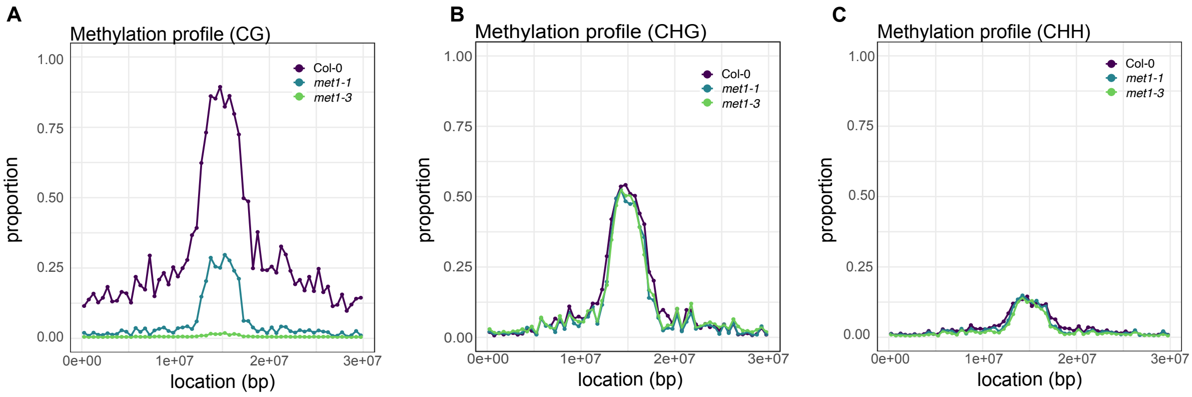

Supplementary Figure S7. *Low resolution (500 Kb) methylation profiles of chromosome 1 in met1-1 and met1-3 mutants.* We considered the methylation contexts individually: *(A)* CG, *(B)* CHG and *(C)* CHH. Black line (Col-0), blue line (*met1-1)* and green line (*met1-3).*

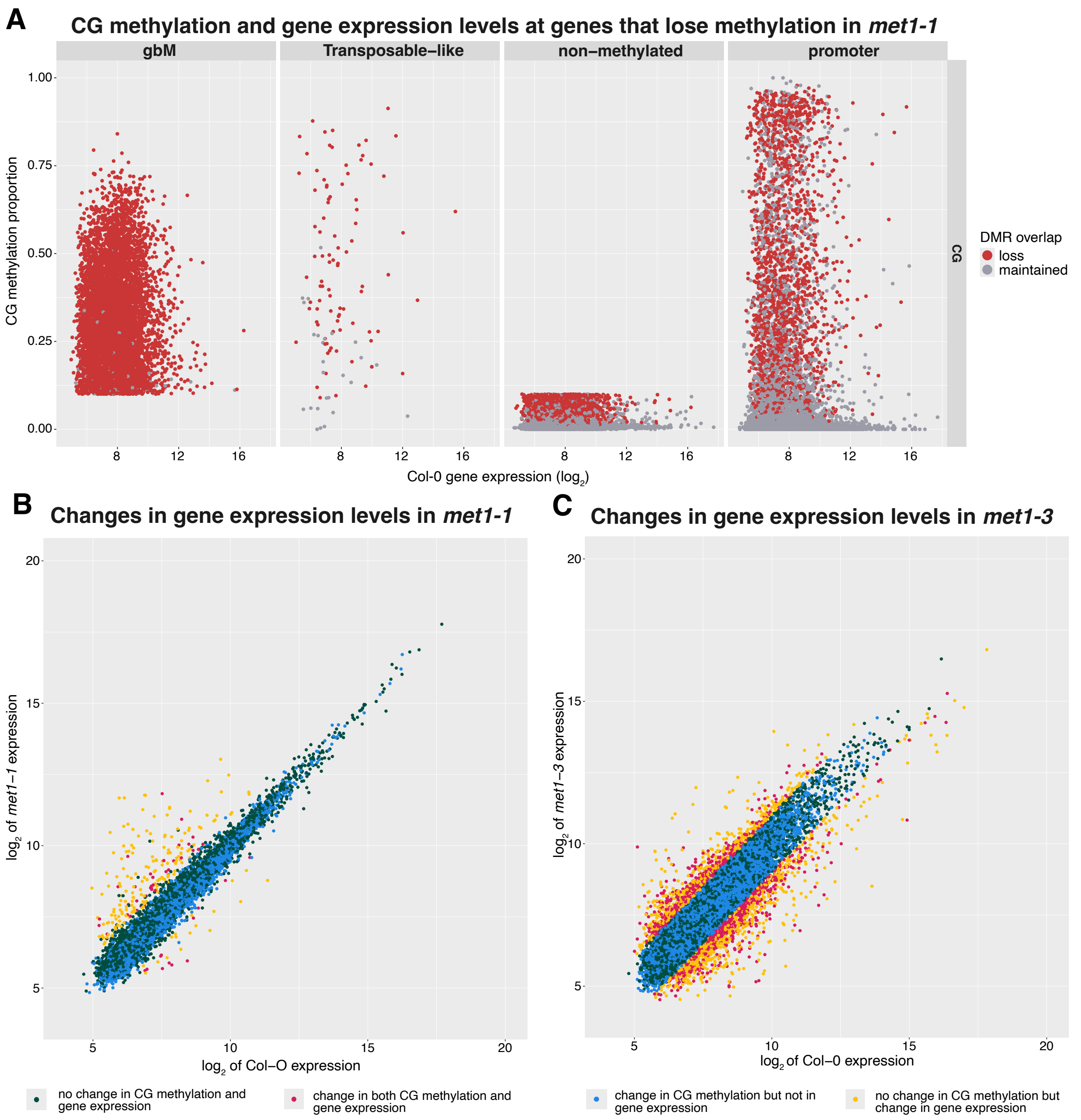

Supplementary Figure S8. *Changes in gene expression in MET1 mutants. (A)* We plot the methylation level and gene expression level for all genes in Col-0 plants. Genes that lose methylation in *met1-1* mutant are highlighted with red. We considered separately the case of gene body methylation (CG methylation proportion higher than 0.1 but CHG and CHH methylation proportion equal to or below 0.05), transposon like methylation (CHG or CHH methylation proportion higher than 0.05), no methylation (CG methylation proportion below or equal to 0.1, and CHG and CHH below or equal to 0.05) and methylation at promoters (CG methylation proportion above 0.1 or CHG or CHH above 0.05). In addition, we consider context specific methylation (CG, CHG and CHH). *(B-C)* Comparison between gene expression levels in *(B)* Col-0 and *met1-1* mutant, and *(C)* Col-0 and *met1-3* mutant. Genes are separated into four groups: no change in methylation and gene expression levels (green); no change in DNA methylation but change in gene expression (yellow), loss of DNA methylation but no change in gene expression (blue); and change in both DNA methylation and gene expression (red).

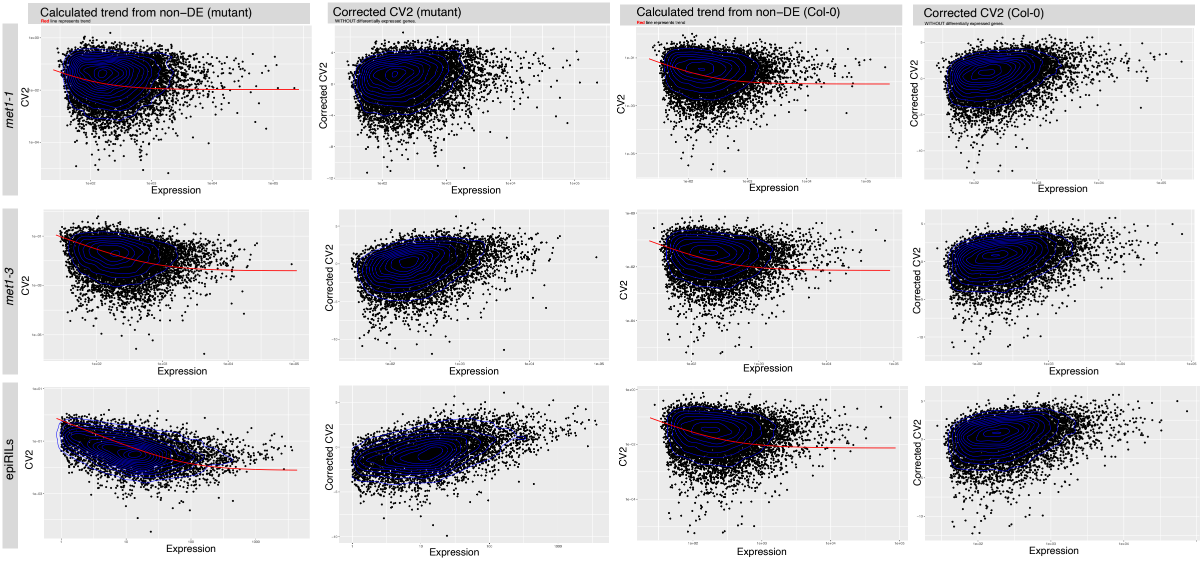

Supplementary Figure S9. *The relationship between gene expression and variability for genes that are not differentially expressed.* Scatterplot of the Coefficient of variation (uncorrected and corrected) as a function of gene expression in the mutant and corresponding Col-0. For details on the computation of corrected CV^2 see *Materials and Methods* section in the main manuscript. We considered the case of: *met1-1, met1-3* and epiRILs.

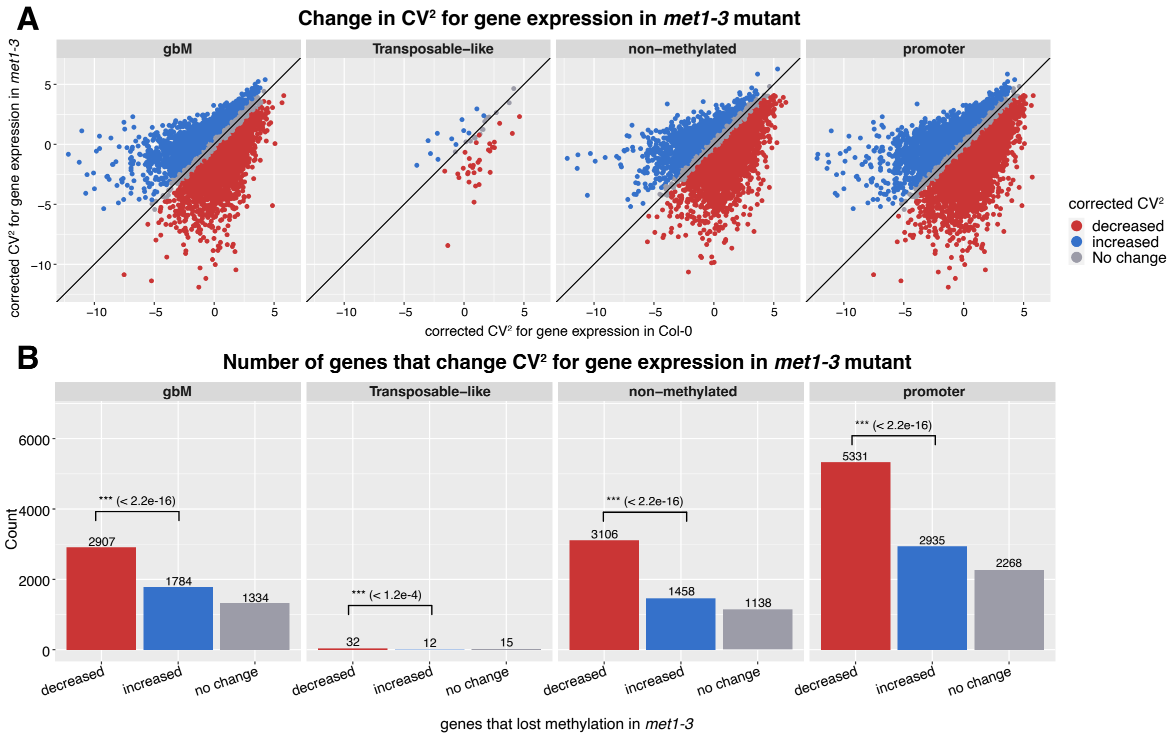

Supplementary Figure S10. *Changes in noise in gene expression in met1-3 mutant. (A)* Comparison between the corrected CV^2 in Col-0 and *met1-3* plants; data from (38). We considered in this analysis only genes without significant change in gene expression between Col-0 and the mutant but displaying loss of DNA methylation (overlap with a hypomethylated DMR in *met1-3)*. Genes are grouped based on their coefficient of variation change: (blue) increased for genes with corrected CV^2 fold change greater than 0.5, (red) decreased for genes with corrected CV^2 fold change less than –0.5, and (grey) no change in corrected CV^2. *(B)* Number of non-differentially expressed genes with loss of DNA methylation between Col-0 and *met1-3* in the different categories shown in panel A.

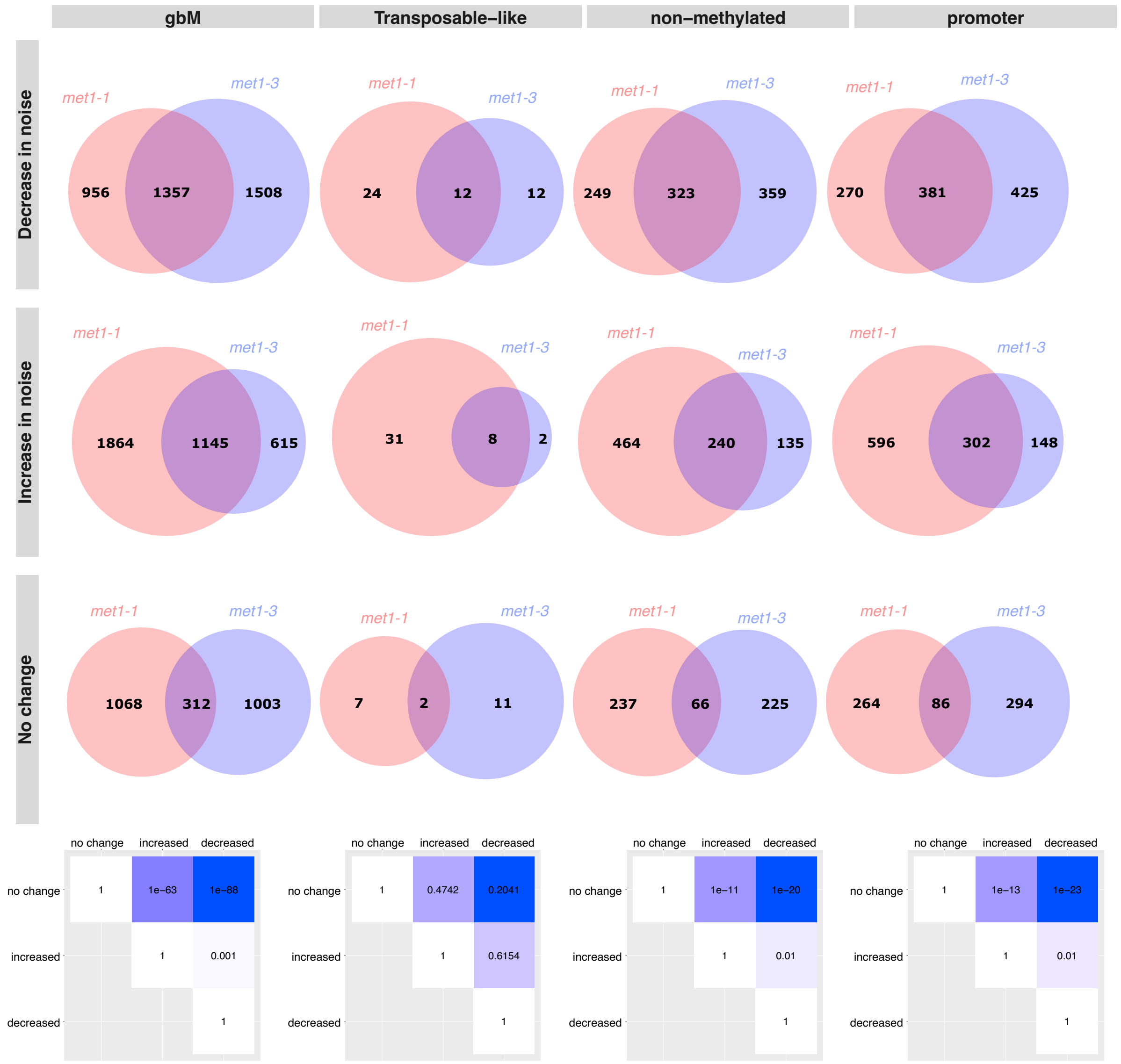

Supplementary Figure S11. C*omparison between changes in noise in gene expression in MET1 mutants. (A)* Venn diagrams comparing the number of gene that decrease, increase or do not change the coefficient of variation in *met1-1* (red) and *met1-3* (blue) mutants. Genes are split into four categories: *(i)* gene body methylated genes, *(ii)* transposon like methylated genes, *(iii)* non-methylated genes and *(iv)* promoter methylated genes. *(B)* Statistical test for the overlaps for each category in panel (A), calculated using Fisher's Exact Test.

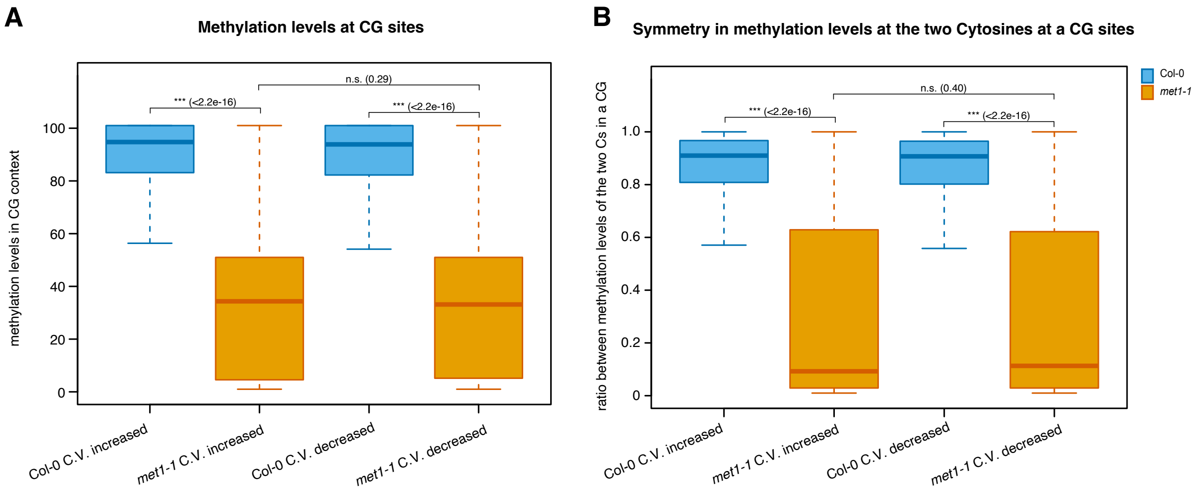

Supplementary Figure S12. *met1-1 mutant displays asymmetry of CG methylation*. *(A)* The methylation levels of all two cytosines in CG context within gene bodies. We considered all CGs that have at least 25% methylation for at least one of the two cytosines in Col-0 plants. We then subset those that overlapped genes that displayed either increase or decrease in variability in gene expression in *met1-1* mutant, while maintaining the same expression levels as in Col-0 (the CG only in Figure 3B from the main manuscript). We also performed the Mann–Whitney U test with corresponding P-value added to the plot. *(B)* Boxplot represents the proportion of CG cytosine pairs with symmetric methylation (< 20% difference between the two cytosines) and the total number of CG site for which at least one cytosine is 25% methylated in Col-0 and *met1-1*. We also performed the Mann–Whitney U test between the symmetry ratio in Col-0 and *met1-1* and between CGs in genes that display increase variability and CGs in genes that display decrease variability in *met1-1* (corresponding P-value added to the plot).

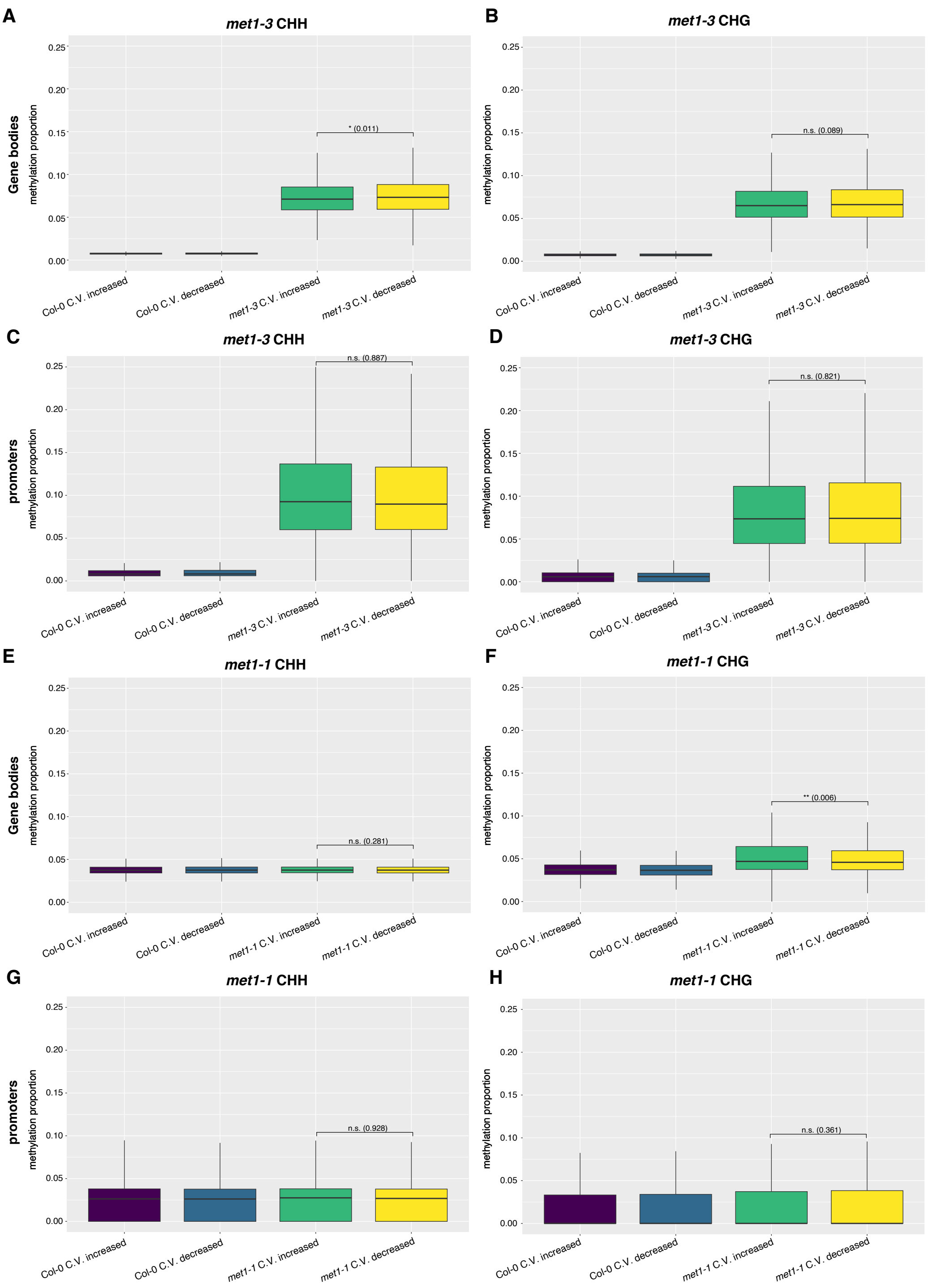

Supplementary Figure S13. *non-CG methylation in met1-1 and met1-3 mutants at genes that display increase or decrease of noise in transcription*. Methylation level of cytosines in: (*A*, *C, E and G*) CHH and (*B*, *D, F and H*) CHG context located (*A-B and E-F*) within genes or (*C-D and G-H*) promoters of genes that displayed an increase or decrease in variability in gene expression in *met1-3* while maintaining the same expression levels as in Col-0. We considered the case of *met1-3* mutant in (A-D) and *met1-1* mutant in (E-H). We performed the Mann–Whitney U test with corresponding P-value added to the plots.

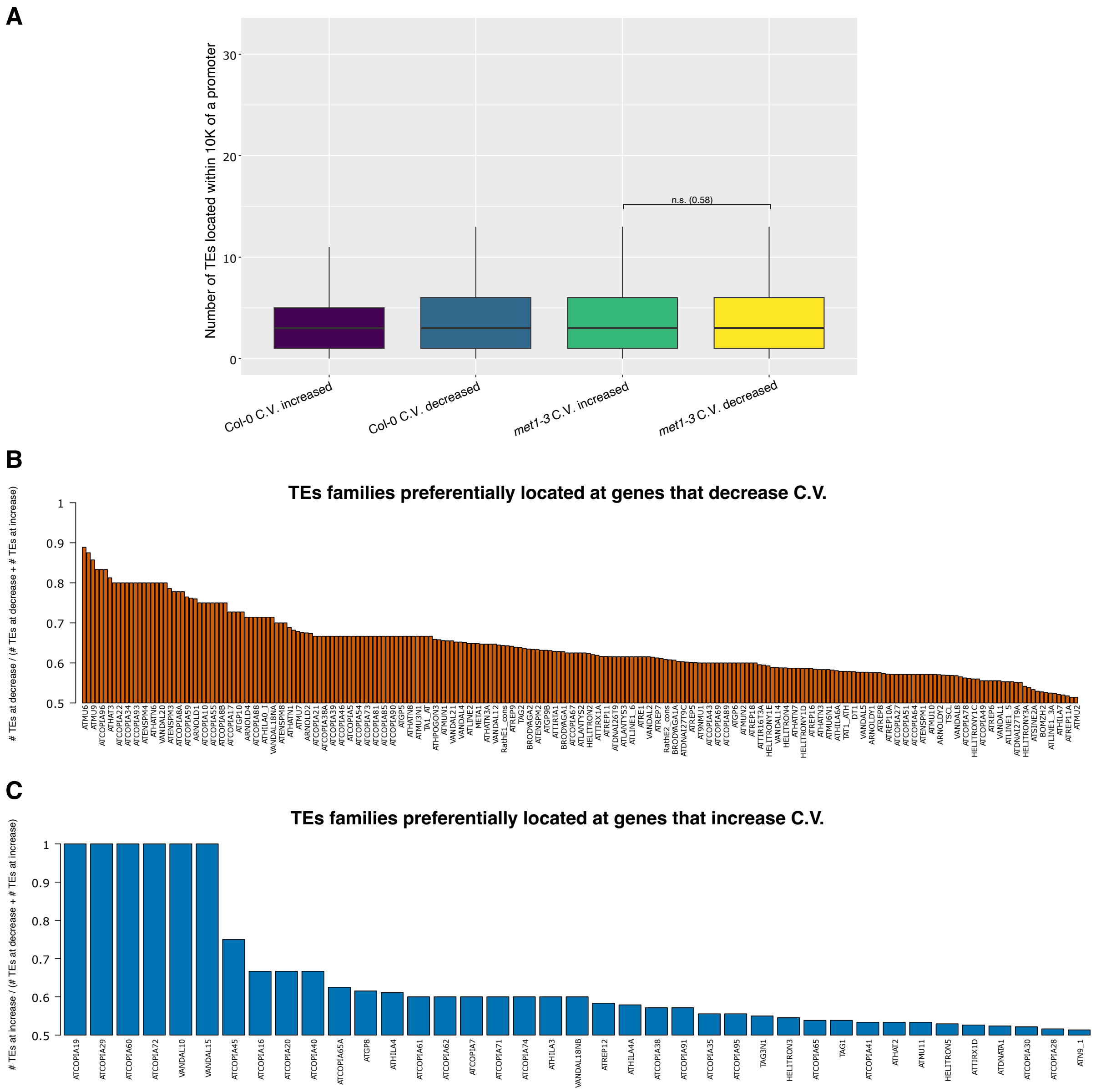

Supplementary Figure S14. *Transposable elements near promoters of genes that display increase or decrease of noise in transcription. (A)* Number of TEs near promoters of genes that displayed an increase or decrease in variability in gene expression in *met1-3* while maintaining the same expression levels as in Col-0. *(B)* Barplot representing the number of copies of different TEs located within 10Kb of a promoter of a gene that displays lower noise in *met1-3*. The plot presents the TE families that have a higher proportion of copies near promoters of genes that display lower noise in *met1-3* (ratio between number of copies near genes that decrease C.V. and number of copies at both genes that increase and decrease C.V.)*. (C)* Barplot representing the number of copies of different TEs located within 10Kb of a promoter of a gene that displays higher noise in *met1-3*. The plot presents the TE families that have a higher proportion of copies near promoters of genes that display higher noise in *met1-3* (ratio between number of copies near genes that increase C.V. and number of copies at both genes that increase and decrease C.V.)*.*

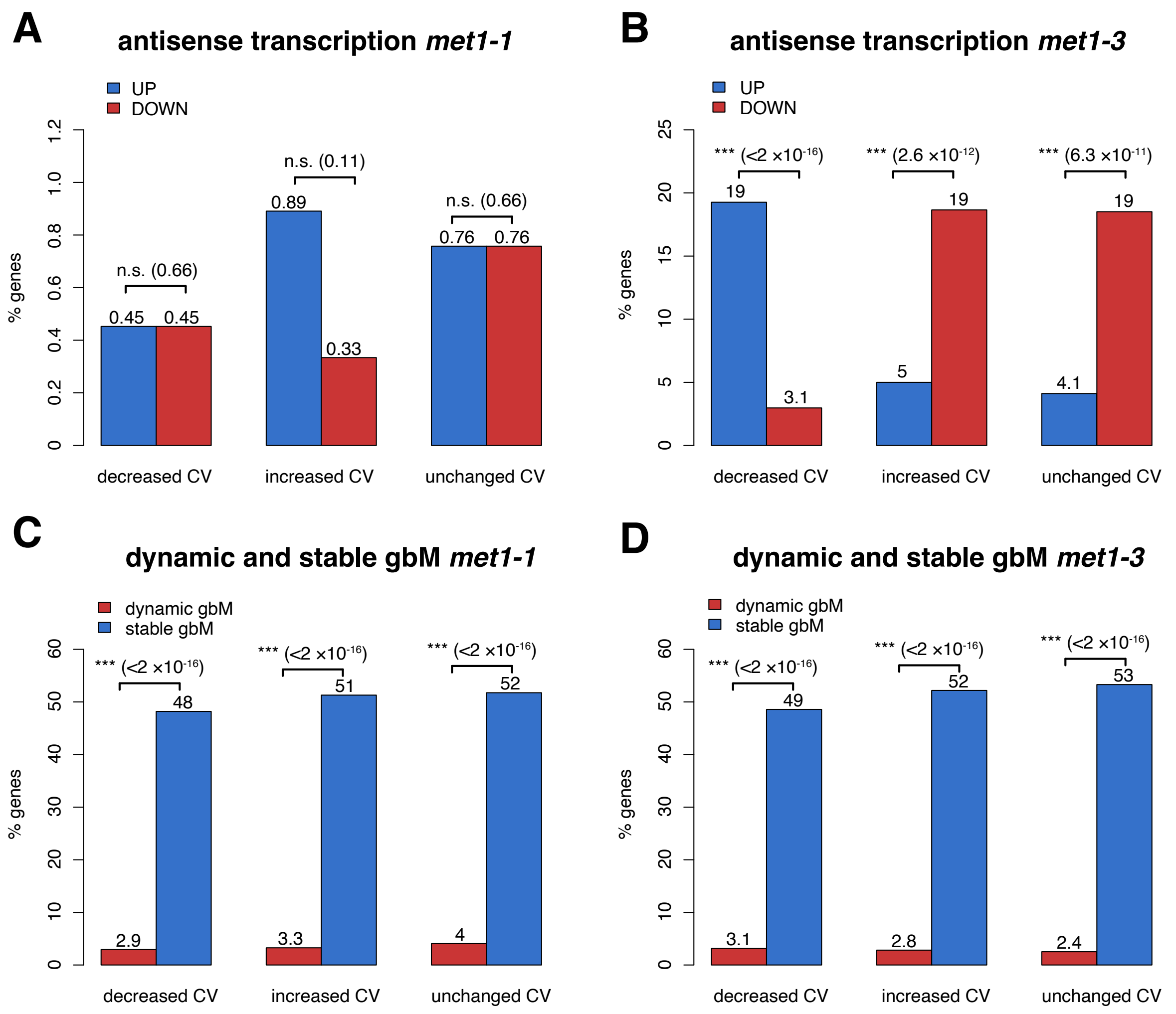

Supplementary Figure S15. *Link between genes that increase noise in met1 mutants and antisense transcription and dynamic vs stable gene body methylation. (A-B)* Percentage of genes that increase, decrease or keep the C.V. unchanged when they overlap with an antisense gene that is either upregulated or downregulated (percentage of the total genes in the corresponding category that have an antisense gene). We considered the case of *(A) met1-1* and *(B) met1-3* separately. *(C-D)* Percentage of genes that increase, decrease or keep the C.V. unchanged separated into genes that have dynamic or stable gene body methylation (percentage of the total genes in the corresponding category). We considered the case of (A) *met1-1* and (B) *met1-3* separately.
